# Exocytosis-coordinated mechanisms for tip growth underlie pollen tube growth guidance

**DOI:** 10.1038/s41467-017-01452-0

**Published:** 2017-11-22

**Authors:** Nan Luo, An Yan, Gang Liu, Jingzhe Guo, Duoyan Rong, Masahiro M. Kanaoka, Zhen Xiao, Guanshui Xu, Tetsuya Higashiyama, Xinping Cui, Zhenbiao Yang

**Affiliations:** 1 Horticultural Biology and Metabolomics Center, Haixia Institute of Science and Technology, Fujian Agriculture and Forestry University, Fuzhou, Fujian, 350002 China; 2 Department of Botany and Plant Sciences, Institute of Integrated Genome Biology, University of California, Riverside, CA, 92521 USA; 3 Division of Biological Science, Graduate School of Science, Nagoya University, Furo-cho, Chikusa-ku, Nagoya, Aichi 464-8601 Japan; 4 Department of Statistics, University of California, Riverside, CA, 92521 USA; 5 Department of Mechanical Engineering, University of California, Riverside, CA, 92521 USA; 6 Institute of Transformative Bio-Molecules (WPI-ITbM), Nagoya University, Furo-cho, Chikusa-ku, Nagoya, Aichi 464-8601 Japan

## Abstract

Many tip-growing cells are capable of responding to guidance cues, during which cells precisely steer their growth toward the source of guidance signals. Though several players in signal perception have been identified, little is known about the downstream signaling that controls growth direction during guidance. Here, using combined modeling and experimental studies, we demonstrate that the growth guidance of *Arabidopsis* pollen tubes is regulated by the signaling network that controls tip growth. Tip-localized exocytosis plays a key role in this network by integrating guidance signals with the ROP1 Rho GTPase signaling and coordinating intracellular signaling with cell wall mechanics. This model reproduces the high robustness and responsiveness of pollen tube guidance and explains the connection between guidance efficiency and the parameters of the tip growth system. Hence, our findings establish an exocytosis-coordinated mechanism underlying the cellular pathfinding guided by signal gradients and the mechanistic linkage between tip growth and guidance.

## Abstract (web summary)

Tip-growing cells can find their growing path toward the source of attractive signals. Here, using experimental data and mathematical modeling, Luo et al. demonstrate that tip-localized exocytosis can integrate guidance cues with Rho GTPase signaling to control cell wall mechanics and direct tip growth in *Arabidopsis* pollen tubes.

## Introduction

The growth of many cells is guided to a long-distance destination along a gradient of external signals to achieve their functions in the delivery of signals or structures, or the exploration of environments. Fungal hyphae are attracted to the sources of nutrients or pheromones. Animal neuronal axons follow precise paths to reach their targets. Plant pollen tubes are guided to the ovules to deliver sperms for fertilization. The mechanisms by which these cells find their targets are poorly characterized, although the guided growth is tightly linked to an extreme form of polar growth known as rapid tip growth, which usually occurs in a random direction in the absence of external cues^1^. However, the mechanism behind this linkage remains enigmatic.

Both tip growth and directional growth of walled cells in fungi and plants depend on a tight spatiotemporal control of the mechanical properties of the cell wall. Computational models suggest that the tip growth of these cells requires a deformable growing domain in the apical wall, which yields to turgor pressure to allow tip expansion^2–5^. This domain is assumed to associate with the intracellular biochemical kinetics and the exocytosis-dependent deposition of new wall components and their modifying factors, which altogether determine the extracellular matrix mechanics at the apex^2,4,6–8^. Indeed, the distribution pattern of exocytic machinery reliably predicts the cell shape in slow-growing fission yeast cells^9^. However, the mechanistic roles of exocytosis in the regulation of rapid tip growth and growth guidance have not been elucidated, neither are the intracellular mechanisms that coordinate the spatiotemporal regulation of exocytosis with the cell wall mechanics.

In *Arabidopsis* pollen tubes, a reciprocal regulation between the apical exocytosis and the ROP1 Rho GTPase-dependent signaling network has been suggested^10,11^. Active ROP1 is distributed as a dynamic apical cap in the plasma membrane (PM) that spatiotemporally coincides with and promotes the apical exocytosis^11–14^. The exocytosis is in turn implicated in the negative feedback regulation of ROP1 signaling by targeting the REN1 RhoGAP (Rho GTPase-activating protein that deactivates ROP1)^15^ as well as in the feed-forward regulation of ROP1 by targeting receptor-like kinases (RLKs) that activate ROP1 through RopGEFs (ROP guanine nucleotide exchange factors)^16–18^. Interestingly, active ROP1 and exocytic vesicles were found to redistribute to the future growth sites prior to pollen tube turning^12,19,20^. These observations hint a linkage of the guidance signals to the exocytosis-ROP1 signaling network, but its role in pollen tube guidance has been difficult to determine because both ROP1 signaling and exocytosis are required for tip growth.

Here we combine mathematical modeling with experimental studies to demonstrate that the exocytosis-ROP1 signaling network, coordinating the cell wall mechanics, provides a mechanistic linkage between pollen tube tip growth and growth guidance, and that the exocytosis-centered mechanism for tip growth is critical for the robustness and efficiency of the pollen tube guidance system.

## Results

### An exocytosis-centered model for tip growth

**Fig. 1:**
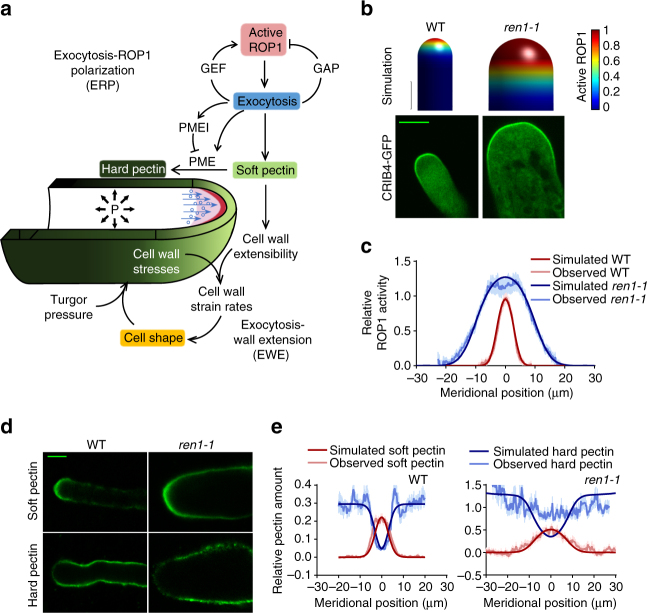
A model of exocytosis-controlled pollen tube tip growth. **a** A conceptual model of pollen tube tip growth. Exocytosis-mediated positive and negative feedback loops generate the apical cap of active ROP1 (red crescent) in the PM. ROP1-dependent exocytosis (blue circles) also delivers soft pectin (light green) to the pollen tube apex, which is converted to hard pectin (dark green) on the shank. The resulted asymmetric cell wall extensibility together with turgor pressure (“P” and black arrows) determines the strain rates and thereby the geometry of the cell wall. **b**–**e** Loss of the ROP1 deactivator, REN1, results in broader active ROP1, broader soft pectin distribution, and wider cell. **b** Simulated and confocal microscopy images of wild-type and *ren1-1 Arabidopsis* pollen tubes expressing CRIB4-GFP that shows the distribution of active ROP1. Only the tip region of pollen tubes was shown. **c** Simulated and observed distribution of active ROP1 in wild-type and *ren1-1* pollen tubes. The observed distribution is the average distribution measured from 24 wild-type or 14 *ren1-1* pollen tubes. **d** Confocal microscopy images of pectin immunostaining of wild-type and *ren1-1* pollen tubes with JIM7 and JIM5. **e** Observed and simulated distribution of pectins in wild-type and *ren1-1* pollen tubes. Error bars show s.e.m. Scale bar: 5 μm

To elucidate the complex roles of exocytosis, we first sought to build a realistic mathematical model for tip growth that allows testable predictions. Our model is based on the conceptual framework depicted in Fig. 1a. We hypothesize that exocytosis is at the heart of the mechanism for rapid tip growth by controlling the self-polarization of active ROP1 GTPase as well as by relaying the polarized ROP1 activity to cell wall mechanics. Thus the model has two interlinked modules: (1) exocytosis-ROP1 polarization module (ERP) and (2) exocytosis-wall extension module (EWE) (Supplementary Note 1).

ERP describes the exocytosis-dependent self-organization of the ROP1 activity at the apical PM (Fig. 1a). Exocytosis is locally activated by active ROP1 via its regulations of the dynamics of the apical actin microfilaments (F-actin)^10,11^. Thus, the rate of exocytosis along the cell periphery, as a function of position and time, is determined by the local concentration of active ROP1. The distribution of active ROP1 is determined by three processes: ROP1 activation, deactivation, and diffusion. Simulations show that spatial regulation of ROP1 activator and deactivator is essential for the polarization of active ROP1 (Supplementary Fig. 1a). Here we hypothesize that ROP1-dependent exocytosis controls the spatial distribution of the ROP1 regulators. In yeast, both exocytosis-dependent and independent feed-forward mechanisms have been proposed to generate the polarization of Cdc42^21–24^. In pollen tubes, exocytosis-independent feed forward regulation of ROP1 has not been shown. The ROP1-dependent exocytosis likely targets RLKs, for example, PRK2 and PRK6, and their potential peptide ligands to the tip^16,18,25,26^. These PM receptors directly interact with the ROP1 activator RopGEFs to activate ROP1^16,26^. Thus, we assume that the rate of ROP1 activation is locally proportional to the rate of exocytosis. Exocytosis also targets the REN1 RhoGAP to the apical PM, which deactivates ROP1 and limits the active ROP1 to the apical cap^15^. Although negative feedback could be either global or local, simulations show that only global inhibition, in which the ROP1 deactivation rate is proportional to the constant representing the basal exocytosis strength, maintains the apical cap (Supplementary Fig. 1a). This agrees with the broader distribution of REN1 at the tip of pollen tubes^15^ compared to the apex-localized RopGEFs^17^ (Supplementary Fig. 1b). This polarization mechanism is reminiscent of the slow diffusing activator and the fast diffusing inhibitor in the Turing-type patterning system^27^.

Because exocytosis also delivers cell wall polymers and their modifying factors to the apical cell surface, we link the ROP1-mediated exocytosis to the structure and mechanics of the cell wall in the EWE module (Fig. 1a). In pollen tubes, the mechanical properties of the apical wall are evidently determined by its major component, pectin^4,28,29^. Methylated pectin is synthesized in the Golgi body, secreted, and inserted into the apical wall^14,30^. The methylated pectin lacks Ca^2+^-mediated crosslinks and is “soft” and extensible^31^. Exocytosis secretes pectin methylesterases (PMEs), which de-esterify methylated pectin^6,28^. The demethylated pectin forms crosslinks through Ca^2+^ bridges and are structurally rigid, or “hard”^31^. The activity of PMEs is regulated by peptidyl inhibitors, which are also secreted by exocytosis^6^. Hence, exocytosis coordinates the secretion of soft pectin and its conversion to hard pectin, which together determine the extensibility of the cell wall that is assumed to maintain a constant thickness. Using the viscoplasticity theory of turgor pressure-driven wall deformation and the previously established equations for the calculation of stresses, strain rates, and the cell geometry^2,32^, our model reproduces pollen tube shape formation and tip growth (Fig. 1b; Supplementary Movie 1; Supplementary Note 1).

Construction of a realistic model with robust predictive powers demands accurate parameterization. We determined the rate of ROP1 lateral diffusion in the PM by fluorescence recovery after photobleaching (FRAP) using *Arabidopsis* pollen tubes expressing GFP-ROP1 (Supplementary Fig. 1c). The strengths of positive and negative feedback loops, *k*_pf_ and *k*_nf_, were not measurable and thus were estimated from the actual distribution of active ROP1 that was determined using CRIB4-GFP as an active ROP1 marker (Supplementary Fig. 2). Using the trial-and-error method, we computed the combinations of *k*_pf_ and *k*_nf_ that allow simulating the active ROP1 distribution. We also developed a statistical algorithm for estimating *k*_pf_ and *k*_nf_^33^, which efficiently screens for the set of parameters that allows simulation of the active ROP1 distribution. The two methods identified nearly identical *k*_pf_ and *k*_nf_ values. The rate of pectin demethylation was estimated by fitting the simulation with the distribution of soft and hard pectin polymers in wild-type *Arabidopsis* pollen tubes immunostained by JIM7 and JIM5 antibodies, respectively^34–36^. Soft pectin preferentially localizes to the growing apex, while the hard pectin is nearly absent from the apex but primarily distributed to the shank of pollen tubes (Fig. 1d). The simulated pectin distribution and pollen tube shape match well with the observations (Fig. 1e). Sensitivity analysis shows that our model is robust to changes in most parameters (Supplementary Table 1).

To validate our model, we analyzed genetic mutants altered in ROP1 activation and apical cell wall composition. Our simulations show that the distribution of active ROP1 is closely linked with the shape of pollen tubes (Supplementary Fig. 3a), as shown in a previous report on the effect of ROP1 overexpression^37^. We further tested this prediction using *ren1-1*, a loss-of-function mutant of the *REN1* RhoGAP^15^. *ren1-1* is completely male sterile and produces club-shaped tubes, with a mean width of 17.5 ± 0.8 μm (± indicates s.e.m., *n* = 30; vs 6.0 ± 0.2 μm in wild-type tubes, *n* = 24) at 2 h after germination (Fig. 1b). The shape of *ren1-1* tubes matches a pollen tube predicted to have an 80% reduction in *k*_nf_. Furthermore, the *ren1-1* tubes had a dramatic expansion of the active ROP1 cap and the apical soft pectin distribution accompanied by the exclusion of hard pectin from the enlarged apex, which all matches the predicted values (Fig. 1c–e). The phenotypes of other reported mutants with defects in ROP1 signaling, including pollen tubes overexpressing RopGEFs^17^, ROP1, constitutive active ROP1 (CA-rop1), or dominant negative ROP1 (DN-rop1)^38^, were also reproduced by our model (Supplementary Fig. 5f).

Reduction in PME or pectin de-esterification rate (*k*_PME_) is predicted to increase the apical region containing soft pectin with reduced amount of hard pectin, generating wider pollen tubes (Supplementary Fig. 3c, e). As expected, *Arabidopsis vgd1-1* pollen tubes lacking a major PME (VANGUARD1)^29^ displayed a much broader apical area containing the soft pectin but depleted of hard pectin, compared to wild-type tubes, while a partially complemented line of *vgd1-1*, *vgd1-1c*, showed similar but alleviated phenotypes (Supplementary Fig. 3d, e). The high soft pectin content may account for the structural instability reflected by the high bursting rate of pollen tubes observed in *vgd1-1* previously^29^. Corresponding to the differences in pectin distribution, *vgd1-1* tubes with the broadest extensible region at the tip are the widest, followed by *vgd1-1c* tubes (Supplementary Fig. 3d). Thus, the computational modeling and experimental data together show that PME plays an important role in the regulation of growth polarity in pollen tubes.

### Exocytosis modulates ROP1 polarity and cell shape

**Fig. 2:**
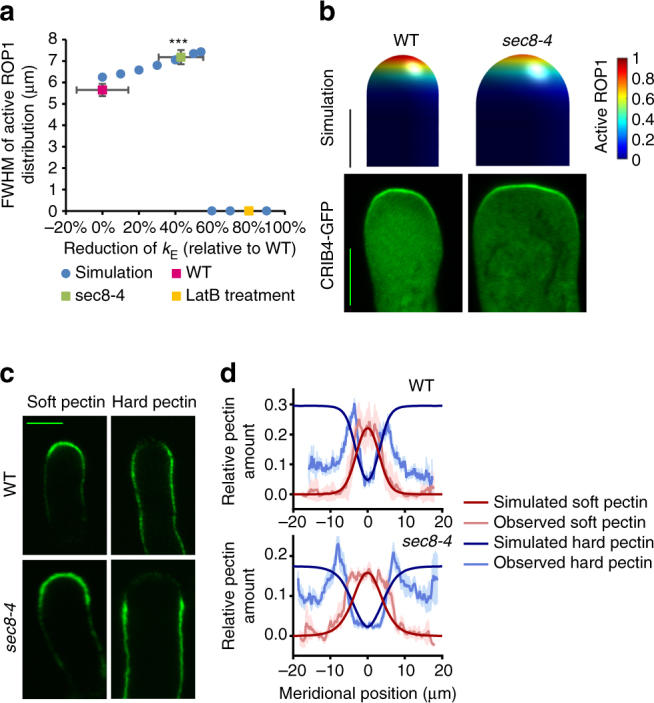
Exocytosis plays a central role in regulating both ROP1 signaling and cell wall mechanics. **a** The distribution of active ROP1 changes with the level of exocytosis. The broadness of active ROP1 distribution is represented by the full width at half maximum (FWHM) of the distribution curves (measured from 24 wild-type and 21 *sec8-4* pollen tubes). The FWHM of active ROP1 increases when exocytosis rate is moderately reduced (by <56%), but drops to 0 (i.e., loss of ROP1 polarity) when exocytosis is severely reduced (by >56%). Circles and squares are results of simulation and measurement, respectively. Error bars show s.e.m. ***P ≤ 0.001 (Student’s *t* test). **b** Simulation and confocal microscopy images of wild-type and *sec8-4* pollen tubes expressing CRIB4-GFP. Only the tip region of pollen tubes was shown. **c** Confocal microscopy images of pectin immunostaining of wild-type and *sec8-4* pollen tubes with JIM7 and JIM5. **d** Observed and simulated distribution of pectins in wild-type and *sec8-4* pollen tubes. Error bars show s.e.m. Scale bar: 5 μm

Due to the complex roles of exocytosis in the feed-forward and feedback regulation of ROP1 signaling as well as cell surface mechanics, it has been difficult to intuitively understand how the system behaves when exocytosis is compromised, but modeling can quantitatively predict the effect of exocytosis (Fig. 2a). Our model predicts a severe reduction in exocytosis, that is, a reduction in *k*_E_ by >56%, will lead to growth cessation resulting from the elimination of the steady-state active ROP1 cap (Fig. 2a) and the growth cessation is preceded by a transient growth depolarization due to the residual but expanded ROP1 activity (Supplementary Fig. 4a). Interestingly, the model predicts a broader distribution of active ROP1, when exocytosis is moderately reduced by <56% (Fig. 2a). In this case, the distribution of soft pectin expands further from the apex, while the hard pectin retreats accordingly leading to wider pollen tubes (Fig. 2d).

We tested these predictions using pollen tubes with different degrees of deficiency in exocytosis, which is measured using corrected fluorescence recovery after photoconversion (cFRAPc) analysis^13^. To achieve a severe reduction in exocytosis, pollen tubes expressing CRIB4-GFP were treated with 50 nM of Latrunculin B (LatB), which promotes actin depolymerization and inhibits exocytosis. In 30 min after LatB treatment, the active ROP1 cap and tip growth were abolished, as the model predicts (Fig. 2a, Supplementary Fig. 4a, b). The model also predicts transient spreading of ROP1 before complete depolarization that results in bulging tips, consistent with the experimental observation (Supplementary Fig. 4a, b). Subapical distribution of active ROP1 following LatB treatment was often observed and may be caused by non-uniform inhibition of exocytosis as shown by the simulation (Supplementary Fig. 4c). In the pollen tubes of *sec8-4*, a weak mutant for an exocyst subunit gene *SEC8* ^39^, exocytosis rate was reduced by 43% (Supplementary Fig. 4d–f). The *sec8-4* tubes show a broader distribution of active ROP1 and soft pectin, a wider region depleted of hard pectin at the tip, and increased tube width (8.4 ± 0.3 μm), consistent with the predicted values given the 43% reduction in exocytosis (Fig. 2a–d). Here a non-saturating concentration of the pectin antibodies revealed a reduction of hard pectin at the shank of pollen tubes, which was likely to be resulted from the replacement of pectin by other wall materials not included in the model, such as callose and cellulose^40^. These results reveal a critical role for exocytosis in the regulation of ROP1 polarity and the shape of tip-growing cells.

### The mechanisms for tip growth control pollen tube guidance

**Fig. 3:**
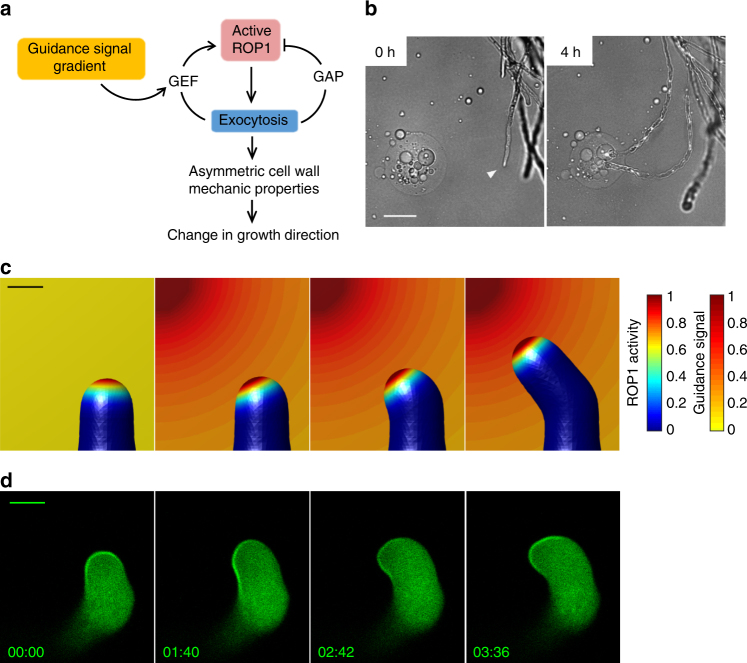
The tip growth system underlies the growth guidance of pollen tubes. **a** Illustration of the signaling part of the guidance model. The external guidance signal promotes ROP1 activation through the positive feedback. A signal gradient leads to an asymmetric distribution of active ROP1 and exocytosis. By regulating the cell wall mechanical properties, asymmetric exocytosis results in the change of growth direction. **b** Observing the growth guidance of *Arabidopsis* pollen tubes using the semi-in vitro assay with AtLURE1-containing gelatin beads. Arrowhead indicates the tip of the pollen tube being observed. Numbers are time after the beads were placed nearby the tip of the pollen tube. Scale bar: 50 μm. **c**, **d** Simulation and observation of active ROP1 redistribution prior to the turning of a pollen tube during growth guidance. **c** Simulation of a pollen tube when exposed to a gradient of guidance signal. Color of the cell surface and the background indicates the level of active ROP1 and the concentration of guidance signal, respectively. Scale bar: 5 μm. **d** Confocal microscopy images showing the turning of a pollen tube expressing CRIB4-GFP attracted by an AtLURE1-containing gelatin bead (upper left to the pollen tube). The images are representative of ten samples. Numbers show time (min:s). Scale bar: 5 μm

Assuming guidance signaling is independent of the ROP1-exocytosis signaling network, e.g., via modulating cell wall mechanical properties directly, simulation shows that the pollen tube becomes depolarized and approaches the source much less efficiently than observed (Supplementary Fig. 5a), since the gradient of the signal itself is not sufficient to sustain highly polarized growth. Therefore, we propose that guidance signals regulate the tip growth machinery: an asymmetrically distributed guidance signal causes the redistribution of active ROP1 and the re-direction of exocytosis, leading to the change in tip growth direction (Fig. 3a). In this model, the local feed-forward rate, *k*_pf_, is positively correlated with the local signal concentration (Fig. 3a). The deformation of the cell wall with asymmetric extensibility is solved numerically using the finite element method (Supplementary Note 1).

In response to guidance signal gradients, the model suggests that the apical active ROP1 shifts to a new position according to the signal source followed by growth turning at that position (Fig. 3c; Supplementary Fig. 5b; Supplementary Movie 2). We validated this prediction by observing the dynamics of active ROP1 in a semi-in vitro guidance assay^41^, in which pollen tubes turned toward dissected ovules or gelatin beads containing purified AtLURE1 guidance signal^42^ placed on one side of the cell tips (Fig. 3b). As predicted, active ROP1 shifting to the signal source was followed by growth reorientation (Fig. 3c, d; Supplementary Movie 3). Furthermore, propidium iodide staining showed asymmetric deposition of cell wall materials preceding the turning of pollen tubes (Supplementary Fig. 5c). The connection of guidance to ROP1 signaling is also consistent with the identification of PM-localized RLKs perceiving the AtLURE1^43,44^. In particular, the PRK6 RLK was found to interact with RopGEFs^43^. Like active ROP1, PRK6 also redistributes toward the source of diffusive AtLURE1, prior to the turning of pollen tubes^43^. Importantly, our model reproduces this redistribution given an exocytosis-based targeting of PRK6 to the PM (Supplementary Fig. 5d). Hence, we conclude that the guidance of pollen tube is a result of the reorientation of the exocytosis-ROP1 signaling network that controls tip growth.

### Signal gradients and growth rates impact guidance efficiency

**Fig. 4:**
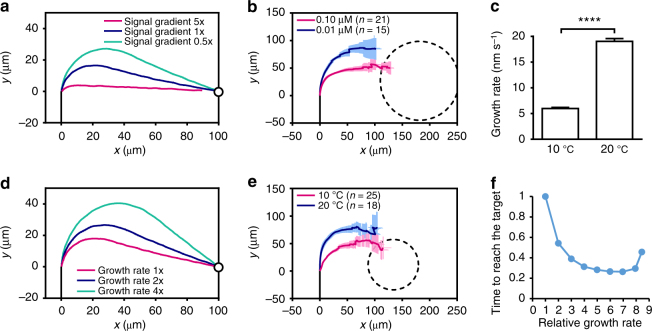
Signal gradients and growth rates impact guidance efficiency. **a**, **d** Simulated trajectories of pollen tubes exposed to a gradient of guidance signal. The tip of pollen tube is at [0, 0] when being exposed to guidance signal. Black lines represent the midline of the pollen tube before being exposed to guidance signal. Circles represent the source of the guidance signal. **b**, **e** Average trajectories of pollen tubes growing toward AtLURE1-containing gelatin beads in the semi-in vitro guidance assay. Dashed lines in show the outline of the gelatin beads. Error bars show s.e.m. *x* and *y*: the horizontal and vertical directions of the plane in which pollen tubes are growing. **a**, **b** Pollen tubes exposed to higher level of signal gradients take shorter paths to the source of signal. In **b**, different levels of signal gradient were generated by gelatin beads containing 0.10 μM or 0.01 μM AtLURE1. **c** Pollen tubes growing at different rates were achieved by incubation at different temperatures: the average growth rate of pollen tubes growing semi-in vitro incubated at 10 °C (*n* = 47) is significantly lower than those at 20 °C (*n* = 46). Error bars show s.e.m. ****P ≤ 0.0001 (Student’s *t* test). **d**, **e** Slower growing pollen tubes make sharper turnings when exposed to the same level of signal gradient. **f** The relationship between the growth rate (ratio to wild-type value) of a pollen tube and the time to reach the target (ratio to wild-type value) given the same guidance signal gradient predicted by the model

We sought to explore modeling-inspired new insights into pollen tube guidance behaviors. Synergid cells in ovules contain highly invaginated PM to increase the surface area for secreting guidance signals. These peptidyl guidance signals are encoded by a family of highly duplicated genes presumably to increase the amount of signals. Thus, it is anticipated that high levels of guidance signals are produced at the micropylar pole to generate steep gradients. Our guidance model predicts that with a steep gradient, the active ROP1 distribution shifts quickly and the pollen tube makes a sharp turning, whereas a shallow signal gradient induces a slower and smoother turning (Fig. 4a). We tested this prediction using the semi-in vitro guidance assay with gelatin beads containing different concentrations of AtLURE1^42^ (Fig. 3b). As predicted, pollen tubes took sharper turns and shorter paths to the beads with higher concentrations of AtLURE1 (Fig. 4b). Therefore, steep signal gradients induce efficient guidance with sharp turns and short paths to the source of the guidance signal.

In our semi-in vitro guidance assay, we frequently observed dramatic slowdown of pollen tube growth right before turning toward the source of guidance signals. This may hint that rapidly growing tubes need to slow down before they can precisely redirect the growth along the signal gradients. Interestingly, modeling shows that a slower tube makes a sharper turn toward the signal source and finds the target by a shorter path (Fig. 4d). To test this prediction, different growth rates were achieved by incubating pollen tubes at various temperatures. The growth rate at 10 °C was about half of that at 20 °C (Fig. 4c, Supplementary Fig. 6a). Change of temperature may affect other cellular processes, but we found no observable difference in cell morphology under these two temperatures (Supplementary Fig. 6b), indicating that the tip growth mechanism was not affected. As predicted, slower tubes growing at 10 °C made much sharper turns compared with the faster tubes at 20 °C (Fig. 4e). As a result of the tradeoff between the growth rate and the path length, there is an optimal growth rate that minimizes the time to reach the target (Fig. 4f). If the growth rate is too fast, the pollen tube may miss the target completely (Supplementary Fig. 6c). These computational and experimental results demonstrate an important role for growth rate control in determining guidance efficiency and explain a need for pollen tubes to slow down growth before turning. The integration of guidance signaling with the tip growth provides an efficient mechanism by which a desired guidance efficiency can be achieved by coupling guidance signaling with the regulation of growth rates.

### Tip growth mechanism and guidance behaviors

We further assessed the significance of the ROP1-exocytosis-mediated linkage of tip growth to guidance. We reason that the tip growth-based mechanism allows high efficiency and robustness in guidance, because the mechanisms for sensing and transducing guidance signals are restricted to the growing tip and involve exocytosis-coordinated feed-forward and negative feedback loops. Indeed, the simulated pollen tube is highly responsive to very shallow gradients of guidance signal, while the tube maintains a normal cell morphology after making a sharp turn in response to extremely steep gradients (Supplementary Fig. 5e), as observed occasionally during the semi-in vitro guidance assay (Supplementary Fig. 5f). These results show that the guidance system is highly robust in a wide range of signal gradients. This robust signaling system may provide the flexibility for optimal guidance in different plant species and under varying environmental conditions.

**Fig. 5:**
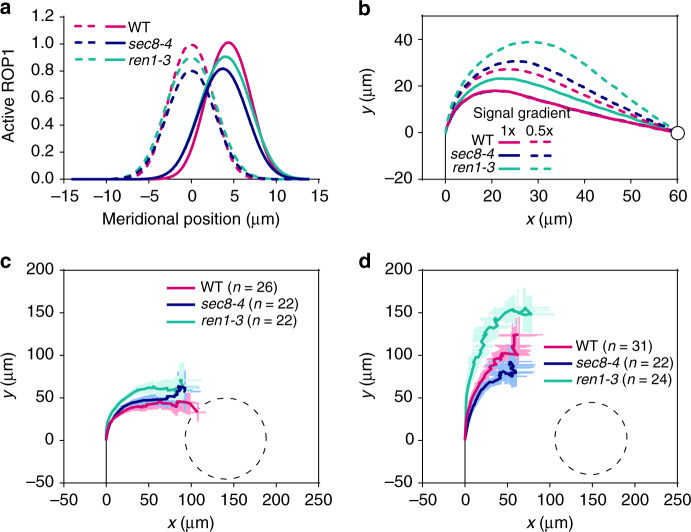
The ROP1-exocytosis signaling is required for sensitive response to guidance signal. **a** The redistribution of active ROP1 in response to guidance signal gradients is slower in *sec8-4* and *ren1-3* than in wild-type (WT). Dashed lines: active ROP1 distribution in the absence of signal gradients; solid lines: active ROP1 distribution after being exposed to signal gradients for a certain period of time. **b** Simulated trajectories of *sec8-4* and *ren1-3* pollen tubes exposed to 1× or 0.5× signal gradient. Lines of wild-type and *sec8-4* at 1× signal gradient are overlapping. The tip of pollen tube is at [0, 0] when being exposed to guidance signal. Black lines represent the midline of the pollen tube before being exposed to guidance signal. Circles represent the source of the guidance signal. **c**, **d** Average trajectories of wild-type, *ren1-3* and *sec8-4* pollen tubes growing toward gelatin beads containing 0.05 μM **c** or 0.02 μM AtLURE1 **d** in the semi-in vitro guidance assay. Dashed lines in show the outline of the gelatin beads. Error bars show s.e.m. *x* and *y*: the horizontal and vertical directions of the plane in which pollen tubes are growing

To assess the significance of the tip growth mechanism in pollen tube guidance, we examined pollen tube guidance of mutants that are compromised in tip growth. Simulations show that in response to signal gradients, active ROP1 redistributes more rapidly in wild-type pollen tubes than in pollen tubes from *ren1-3*, a weak mutant of *REN1* simulated by reducing the negative feedback rate of ROP1 activation by 30%, or *sec8-4*, the mutant with exocytosis rate reduced by 43% (Fig. 5a). As a result, *ren1-3* pollen tubes make slower and smoother turnings to the guidance signal, and reach the target more slowly than wild-type pollen tubes, particularly when the guidance gradient is shallow (Fig. 5b). In the *sec8-4* mutant, however, the growth rate of pollen tubes is reduced, as shown by both simulation and measurement (Supplementary Fig. 6d). When both the ROP1 redistribution rate and the growth rate are taken into account, simulated growth trajectories of *sec8-4* and wild-type tubes are similar (Fig. 5b), but *sec8-4* tubes take longer time to reach the target due to the slower growth rate and thus are less efficient in guidance than wild-type pollen tubes.

To experimentally test these predictions, we measured the trajectories of *ren1-3* and *sec8-4* mutant pollen tubes in the LURE-beads assay. The turning of the average trajectory of wild-type pollen tubes was earlier than that of the *ren1-3* pollen tubes, and as also predicted by the model, this difference was much greater in the presence of a shallower guidance signal gradient (Fig. 5c, d). Furthermore, consistent with the model, there was no significant difference in turning trajectory between *sec8-4* and wild-type in response to either high or low guidance signal gradients, despite the fact that *sec8-4* pollen tubes grew more slowly than wild-type tubes (Fig. 5c, d). These results confirm the predicted role of exocytosis in controlling ROP1 redistribution. The slight inconsistency between the predicted and measured trajectories for wild-type and *sec8-4* pollen tubes likely reflects the quantitative imprecision of our model, as our model is not able to predict precisely how much the growth rate and how much the rate of ROP1 redistribution affect the guidance trajectory, respectively. Furthermore, robust guidance response to a wide range of signal gradients also relies on the ROP1-exocytosis signaling. In response to 0.02 μM AtLURE1, there was a much higher probability for *ren1-3* and *sec8-4* pollen tubes to completely miss the target ((22.6% (7/31) and 18.5% (5/27), respectively)), comparing to wild-type tubes (8.8% (3/34)). Hence, we conclude that the integration of growth guidance with the tip-growth signaling network allows pollen tubes to achieve robust and efficient guidance.

## Discussion

The molecular mechanisms underpinning the targeting of elongating cells to a long-distance destination have been under intensive scrutiny, but how these cells are precisely guided over a long range and why rapid tip growth is necessary for guidance remain enigmatic. In the current study of *Arabidopsis* pollen tubes, we constructed and experimentally validated an integrative mathematical model that underlies both intrinsic tip growth and directed growth guided by gradients of diffusible signals. Our findings established an exocytosis-centric design principle for the linkage between rapid tip growth and growth guidance and demonstrated that this principle provides a crucial mechanism for efficient guidance sensing and signaling in pollen tubes. Therefore, our work here has produced the first mechanistic framework underlying pollen tube guidance by linking guidance signals through the signaling network to cellular and mechanical processes necessary for growth reorientation.

Our studies here show that a centralized coordination of various processes by exocytosis is essential in a rapid tip growth system. In cells without fast tip growth such as budding yeast, Rho GTPase (Cdc42) polarization can be achieved by reaction-diffusion Turing-type mechanism, which can be independent of local exocytosis-based positive feedback^22,45,46^. However, in cells that undergo fast tip growth where the growing end is far away from the geometrical center of the cell, the Rho GTPases, their regulators and downstream targets, and the deposition of cell surface materials need to be highly coordinated to ensure sustained local extension. By integrating computational and experimental analyses, we demonstrate that exocytosis is a central process coordinating these events and thereby participates both in maintaining the polarized active ROP1 cap and regulating the mechanical properties of cell wall. We have constructed a mathematical model that is based on this exocytosis-centered mechanism, and our model reproduces the phenotypes of various mutants defected in ROP1 signaling or cell wall components. Therefore, our model provides a mechanistic, quantitative, and predictive understanding of dynamic cell polarity regulation behind rapid tip growth in pollen tubes with regard to interconnected key processes involved, such as ROP1 signaling, exocytosis and cell wall mechanical changes.

Why long-distance growth guidance requires rapid tip growth has been a longstanding question. It has been difficult to address because genetic defects in tip growth nullify growth guidance. Using modeling-inspired approaches, we demonstrated that the exocytosis-centered mechanisms for rapid tip growth are critical for pollen tube guidance. Our model mechanistically unifies the recent findings that PRK6 participates in sensing AtLURE1, directly interacts with ROPGEFs that activate ROP1, and exhibits repositioning in response to AtLURE1^43^. Importantly, our guidance model provides new insights into the mechanisms of growth guidance. First of all, it shows that the ROP1-exocytosis signaling allows pollen tubes to sense a wide range of guidance signal levels while maintaining a highly polarized shape. Secondly, we found that efficient guidance requires an optimal tip growth rate. The integration of guidance signals with tip growth may provide a mechanism for their regulation of growth rates, which is consistent with the function of PRK6 in both pollen tube growth and guidance^43^. Future studies should determine how growth rate is regulated during growth guidance. Finally, analyses of mutants with defects in the ROP1 signaling or exocytosis reveals that the parameters in the ROP1-exocytosis system are fine-tuned to achieve the maximum guidance efficiency.

In other systems such as plant root hairs, fungal hyphae, and animal neuron axons, polarized activation of Rho-family GTPases at the growing tips is also required for tip growth^47–50^. A striking parallel is the regulation of axon guidance by Rac GTPases, which are closely related to ROPs^47,50^. Furthermore, all tip growing cells require targeted exocytosis for growth, and evidence suggests that some signaling molecules may be targeted to the cell apex via exocytosis^51,52^. In other walled tip-growing cells such as fungi, exocytosis also plays a key role in the regulation of cell wall mechanics^53^. Our model may provide a paradigm for the study of rapid tip growth and chemotactic growth of these systems.

The current model is a simplified framework of various pathways that orchestrate pollen tube growth and guidance. Other pathways and mechanisms such as endocytosis, calcium, and phosphoinositide signaling have been shown to play important roles in the tip growth of pollen tubes and other systems^1,54,55^. Computational analysis also showed the limitations of the current model, for example, in contrast to other polarization systems where positive and negative feedbacks contribute to noise filtering^56,57^, the exocytosis-mediated feedbacks do not significantly enhance the robustness of pollen tube guidance against noise (data not shown), suggesting that additional mechanisms are needed for noise buffering in such rapid tip-growing and reorienting systems. Deciphering how the other mechanisms involved in pollen tube growth and guidance are mechanistically linked to the exocytosis-centered framework presented here should be an important future direction.

## Methods

### Mathematical modeling and simulation

Formulation of the models is presented in Supplementary Note 1: mathematical modeling. The models were computationally implemented in the MATLAB software.

### Plant materials

*Arabidopsis thaliana* Columbia-0 ecotype was used as the wild type. Transgenic *Arabidopsis* lines were generated using the *Agrobacterium*-mediated floral-dip method. Plants were grown at 22 °C in growth rooms under a light regime of 16 h of light and 8 h of dark. *ren1-1* and *ren1-3* were described previously^15^. *sec8-4* (SALK 118129) and *vgd1-1* (SALK_011816) were obtained from the SALK Institute.

### Plasmids and transgenic plants

The coding DNA sequence (CDS) for a fragment of RIC4 (AT5G16490) containing the CRIB domain (CRIB4, amino acid 64-130 of RIC4) was fused with the GFP CDS at the C-terminus and subcloned into a binary vector, pCL, which was constructed by inserting the pollen tube specific LAT52 promoter into pCAMBIA1300 using restriction sites SalI and XbaI. The pCL-PRK1-Dendra2 construct was described previously^13^. GFP-ROP1 described previously^38^ was subcloned into pCL. The CDS of SEC8 kindly provided by Dr. J. Fowler^39^ was fused with GFP at the N-terminus and subcloned into pCL to make the pCL-GFP-SEC8 construct, which was introduced into *sec8-4* for complementation. The CDS of VGD1 was fused with GFP at the C terminus and subcloned into the pCL vector to generate the pCL-VGD1-GFP construct, which was introduced into *vgd1-1* for complementation.

### Analyzing pollen tube growth in vitro

*Arabidopsis* pollen tubes were germinated on a solid medium containing 18% (w/v) sucrose, 0.01% (w/v) boric acid, 1 mM CaCl_2_, 1 mM Ca(NO_3_)_2_, 1 mM MgSO_4_, and 0.5% (w/v) agar. Pollen tubes were incubated at 28 °C for 3–4 h before observation under a microscope. For LatB treatment of *Arabidopsis* pollen tubes, LatB dissolved in DMSO was added to a liquid *Arabidopsis* pollen tube medium (same as the solid medium except without agar) to a final concentration of 50 nM. The liquid medium with LatB was then added to pollen tubes 45 min before observation.

### Semi-in vitro guidance assay

Methods were adapted from previous studies^41^. *Arabidopsis* flowers emasculated 1 day before experiments were pollinated and kept in the growth room for 2–3 h. Stigmas were then cut off with a scalpel, placed horizontally on the surface of a solid pollen tube medium, and incubated at 28 °C for 2–3 h. Pollen tubes germinated and penetrated the stigmas that will emerge from the cut end of the stigmas and grow on the medium. To attract pollen tubes, gelatin beads (5% (w/v) gelatin from bovine skin (Sigma-Aldrich) in liquid pollen tube medium, prepared by emulsification in silicone oil, with diameters around 100 μm) containing 0.1 μM (if not specified) His-tagged AtLURE1.2 peptide (prepared in the Tetsuya Higashiyama laboratory) were placed about 150 μm to the tip of pollen tubes.

To measure pollen tube trajectories in the semi-in vitro guidance assay, the size and the position of the AtLURE1 beads were strictly controlled to ensure that they were the same in different experimental groups (statistical analysis was performed to exclude the outliers). Bright field images of pollen tubes were taken with a Nikon Microphot-FXA microscope. Image quantification was performed using the ImageJ software (version 1.45 s). A segmented line was drawn along the trajectory of the cell to obtain its coordinates (the position of the pollen tube tip and the direction of growth when the AtLURE1 bead was placed are the coordinate origin and the direction of the vertical axis, respectively). The trajectory of a pollen tube is described by [*x*(*s*), *y*(*s*)], where *x* and *y* are the horizontal and vertical coordinates on the surface of the medium, which are both functions of *s*, the length of growth. The average position of the tips of *N* pollen tubes, [*x*_0_, *y*_0_], after growing for a length of *s*_0_, is computed by

$$x_0 = \frac{1}{N}\mathop {\sum}\limits_{i = 1}^N {x_i(s_0)} ,y_0 = \frac{1}{N}\mathop {\sum}\limits_{i = 1}^N {y_i(s_0)} $$

where [*x*_*k*_(*s*), *y*_*k*_(*s*)] is the trajectory of the *k*th pollen tube. All experiments were repeated at least for four times.

To observe pollen tubes growing under the semi-in vitro condition with a confocal microscope, the pollen tubes were cultured on a medium on the surface of a cover slide. Before imaging, the cover slide was placed upside down on a glass slide with a concavity to prevent disturbance of the pollen tubes.

### Confocal microscopy and image analysis

Pollen tubes expressing fluorescently-labeled protein were imaged by a Leica SP2 or Leica SP5 laser scanning confocal microscope. Imaging settings: 488 nm excitation and 500–535 nm emission for green fluorescent protein (GFP); 514 nm excitation and 525–640 nm emission for yellow fluorescent protein (YFP); 458 nm excitation and 465–490 nm emission for cyan fluorescent protein (CFP); 543 nm excitation and 555–664 nm emission for mCherry fluorescent protein; 488 nm excitation and 500–540 nm emission for fluorescein isothiocyanate (FITC); 543 nm excitation and 555–620 nm emission for tetramethylrhodamine isothiocyanate (TRITC); 488 nm excitation and 550–750 nm emission for propidium iodide. The median planes of pollen tube tips were taken for analyzing the distribution of fluorescent signals. Image quantification was performed using ImageJ. A segmented line was drawn along the cell periphery to measure the distribution of fluorescent signal on the PM.

### FRAP analysis

FRAP analysis was performed to measure the diffusion constant of proteins in the PM. GFP-ROP1 overexpression pollen tubes were treated with 20 nM LatB for 10–15 min to suppress F-actin-dependent exocytosis. GFP-ROP1 signal in a small region at the apical PM was photobleached with 100% laser power (488 nm), and time-lapse videos were taken with 6 s time intervals between frames. The distribution curves of GFP-ROP1 were used to fit with the diffusion equation of protein in the PM to estimate the diffusion constant.

### cFRAPc analysis

The exocytosis rates of PRK1-Dendra2 in pollen tubes were measured using cFRAPc^13^. The green form of Dendra2 was excited by 488 nm laser with 5% laser power and detected at 500–535 nm; the red form was excited by 543 nm laser with 40% laser power and detected at 555–620 nm. Photoconversion of Dendra2 was performed by scanning the region-of-interest with 3% ultraviolet laser twice. Post-conversion time-lapse videos were taken immediately after photoconversion, with 5.16 s time interval between frames. Data analysis was performed in MATLAB software.

### CRIB4-GFP colocalization and FRET analysis

For the colocalization analysis of CRIB4-GFP with mCherry-RIC4ΔC and the fluorescence resonance energy transfer (FRET) analysis of CRIB4-GFP with CFP-ROP1 and CFP-DNrop1, the CRIB4 fragment was subcloned into pLat52, a vector for transient expression described previously^58^, to generate the pLat52-CRIB4-YFP construct. The pLat52-CFP-ROP1 and the pLat52-CFP-DNrop1 constructs were obtained by replacing GFP in pLat52-GFP-ROP1 or pLat52-GFP-DNrop1 described previously^38^ by CFP. The pLat52-mCherry-RIC4ΔC construct was generated by replacing the GFP of GFP-RIC4ΔC^12^ by mCherry.

Plasmids were transiently expressed in tobacco (*Nicotiana tabacum*) pollen tubes by particle bombardment. Tobacco plants were grown at 25 °C in growth rooms under a light regime of 12 h of light and 12 h of dark. Pollen grains were bombarded with DNA-coated gold particles (0.5 mg particles coated with 1–2 μg plasmids) using a PDS-1000/He particle delivery system (Bio-Rad Laboratories). Bombarded pollens were cultured in a liquid medium containing 18% (w/v) sucrose, 0.01% (w/v) boric acid, 5 μM CaCl_2_, 5 μM Ca(NO_3_)_2_, and 1 mM MgSO_4_ at 28 °C for 3–4 h before observation under a microscope.

For FRET analysis, pLat52-CFP-ROP1 or pLat52-CFP-DNrop1 was coexpressed with pLat52-CRIB4-YFP in tobacco pollen tubes, while pollen tubes expressing pLat52-CRIB4-YFP, pLat52-CFP-ROP1, or pLat52-CFP-DNrop1 alone served as controls to calculate the bleed-through ratio. CFP and YFP images were acquired using the settings specified above. FRET signals were acquired using 458 nm for excitation and 525–640 nm for emission with the sequential scanning mode. The FRET index normalized by the intensity of the donor was calculated using the ImageJ plug-in “FRET and Colocalization Analyzer”^59^.

### Pectin immunostaining and propidium iodide staining

For immunostaining of methylated pectin and demethylated pectin in *Arabidopsis* pollen tubes, pollen tubes grown on a solid medium were treated with fixative (4% formaldehyde freshly prepared from paraformaldehyde, 3 mM MgSO_4_, 2 mM CaCl_2_, 18% Sucrose, 50 mM PIPES buffer, pH 6.9) for 1 h. After washing with PBS (phosphate-buffered saline), pollen tubes were incubated with purified JIM5 and JIM7 polyclonal antibodies^34–36^ (1:300 and 1:600 dilution in PBS, respectively) at 4 °C over night. After washing with PBS, pollen tubes were incubated with the secondary antibody, FITC-conjugated rabbit anti-rat IgG (Sigma F1763), or TRITC-conjugated goat anti-rat IgG (Santa Cruz 3827. 1:250 dilution in PBS for JIM5 and 1:500 dilution for JIM7) at room temperature for 2 h. After washing with PBS, pollen tubes were observed under a confocal microscope. All pectin immunostaining experiments were repeated for three times. Sample size in each related figure: (1) Fig. 1e—soft pectin: wild type *n* = 12, *ren1–1 n* = 10; hard pectin: wild type *n* = 12, *ren1-1 n* = 11, (2) Fig. 2d—soft pectin: wild type *n* = 11, *sec8-4 n* = 6; hard pectin: wild type *n* = 13, *sec8-4 n* = 11, and (3) Supplementary Fig. 3d—soft pectin: wild-type *n* = 20, *vgd1-1c n* = 18, *vgd1-1 n* = 20; hard pectin: wild type *n* = 18, *vgd1-1c n* = 17, *vgd1-1 n* = 18.

To visualize pectin in living pollen tubes, semi-in vitro pollen tubes were growing on medium containing 40 µM propidium iodide^60^. The fluorescence of propidium iodide was excited with 488 nm laser and collected between 550–750 nm with a confocal microscope every 5 s using a ×40 water lens.

### Sensitivity analysis of model parameters

A local parameter sensitivity analysis was performed to assess the robustness of the model^61^. The width of the simulated pollen tube, *W*, was used as the model output in the analysis. *S*_*Wp*_, the sensitivity of *W* to perturbation of a parameter *p* around its nominal value, is defined as

$$S_{Wp} = \frac{{\partial W/W}}{{\partial p/p}}$$

The absolute value of *S*_*Wp*_ represents the level of sensitivity. The sign of *S*_*Wp*_ indicates whether *W* is positively or negatively correlated with a positive change of *p*.

### Statistical analysis

GraphPad Software was used to perform unpaired two-tailed Student’s *t* tests (with Welch’s correction if the s.d. of the two groups are unequal based on *F* test). All data are expressed as mean ± s.e.m. No statistical methods were used to predetermine sample size. Sample sizes were chosen to reach statistical significance (*P* < 0.05).

### Code availability

Pseudocodes of the mathematical model are included in Supplementary Note 1: mathematical modeling. The original MATLAB codes are available from the corresponding author upon request.

### Data availability

All data that support the findings of this study are available from the corresponding author upon request.

## Electronic supplementary material

Supplementary Information

Description of Additional Supplementary Files

Supplementary Movie 1

Supplementary Movie 2

Supplementary Movie 3
