# Supplementary material for: Exocytosis-coordinated mechanisms for tip growth underlie pollen tube growth guidance
Source: Nat Commun. 2017 Nov 22;8:1687. doi: 10.1038/s41467-017-01452-0 (PMC5698331; doi:10.1038/s41467-017-01452-0)
Supplement: Supplementary file 1 — Supplementary Information [file 41467_2017_1452_MOESM1_ESM.pdf]

Supplementary Note 1
Mathematical Modeling

The Model of Pollen Tube Tip Growth

The model of pollen tube tip growth consists of two parts: the Exocytosis-ROP1 Polarization (ERP) module and the Exocytosis-Wall Extension (EWE) module.

I. Exocytosis-ROP1 Polarization

The definitions and values of the parameters in this module are listed in Supplementary Table 2.

The exocytosis rate, *E*, as a function of the meridional position on the plasma membrane (PM), *s* (Supplementary Fig. 1d), and the time, *t*, is determined by the local concentration of active ROP1 in the PM, *R*:

$$E = k_{\mathrm{E}} R^{\alpha}, \tag{1-1}$$

where k_{E} is a constant linking ROP1 activity to the local rate of exocytosis and is determined by factors independent of ROP1, such as the abundance of exocytosis machinery. α is the degree of nonlinearity.

The spatiotemporal dynamics of active ROP1 is determined by three processes: ROP1 activation, deactivation and diffusion:

$$\frac{\partial R}{\partial t} = k_{\mathrm{a}} R_{\mathrm{in}} \left(1 - \frac{R}{R_{max}}\right) - k_{\mathrm{d}} R + D \frac{\partial^2 R}{\partial s^2}, \tag{1-2}$$

Slow lateral diffusion in the PM is an intrinsic property of ROP1 itself as a PM-associated protein. The amount of activated ROP1 per unit time is proportional to the amount of inactive ROP1 (R_{in}), the rate of ROP1 activation (k_{a}), and is saturated to the local highest ROP1 possible (R_{max}); while the reduction of active ROP1 per unit time is determined by the concentration of active ROP1 and the ROP1 deactivation rate (k_{d}). The inactive form of ROP1 (R_{in}) is maintained as a cytosolic pool by RhoGDIs, thus is expressed as a term independent of the membrane location:

$$R_{\mathrm{in}} = R_{\mathrm{total}} - \int R \, \mathrm{d}s, \tag{1-3}$$

The coefficients k_{a} and k_{d} are determined by the local activity of GEF and GAP, which are dependent on the exocytosis rate. In our hypothesis, the polarization of ROP1 is due to local activation and global inhibition, therefore, k_{a} and k_{d} are proportional to the local or global rate of exocytosis, respectively:

$$k_{\mathrm{a}} = k_{\mathrm{pf}} E, \tag{1-4}$$

$$k_{\mathrm{d}} = k_{\mathrm{nf}} k_{\mathrm{E}}, \tag{1-5}$$

k_{pf} and k_{nf} are exocytosis-independent constants determined by the enzyme activity and expression levels of GEF and GAP, respectively. k_{d} is a constant because GAP is assumed to be uniformly distributed in this model. The value of k_{d}, i.e., the concentration of GAP on the PM, however, is proportional to k_{E}, which represents the basal level of exocytosis.

The initial conditions of ROP1 is an arbitrary local perturbation at *s* = 0. For example,

$$R = \begin{cases} 0.3, s \in [-2.5, 2.5] \\ 0, else \end{cases}$$

When the magnitude of the perturbation exceeds a threshold, ROP1 will polarize with the same steady state distribution regardless of the shape of its initial distribution. ROP1 reduces to 0 if the initial perturbation is below the threshold. Random fluctuation of ROP1 can also generate a stable peak of ROP1 at a random location. The boundary condition of ROP1 is $R = 0$ at $s = \pm 30$.

Alternative modes of regulation by exocytosis on ROP1 activity have been tested (Supplementary Fig. 1a): 1) there is no feedback regulation between exocytosis and ROP1, so that both k_a and k_d are constants, and 2) exocytosis regulates ROP1 by local activation and local inhibition:

$$k_a = k_{pf}E, \; k_d = k_{nf}E$$

Simulation reveals that in these two scenarios, active ROP1 is not able to polarize.

II. Exocytosis-Wall Extension

The definitions and values of the parameters in this module are listed in Supplementary Table 3.

(i) Pectin Distribution and Cell Wall Extensibility

The soft pectin is delivered to the cell wall via exocytosis and converted to hard pectin by PME; both soft and hard pectins are affected by local cell wall elongation. Therefore, the distributions of soft and hard pectins are described by

$$\frac{\partial P_s}{\partial t} = k_{sec}E - k_{pme}C_{EP}P_s - \frac{\partial(v_t P_s)}{\partial s}, \quad (1\text{-}6)$$

$$\frac{\partial P_h}{\partial t} = k_{pme}C_{EP}P_s - \frac{\partial(v_t P_h)}{\partial s}, \quad (1\text{-}7)$$

where v_t is the local tangential growth velocity of the cell wall (Supplementary Fig. 1d), and C_{EP} is the concentration of effective PME. PME is evenly distributed on the cell membrane with the concentration proportional to the level of exocytosis at the tip of pollen tubes (E_t):

$$C_{PME} = E_t \quad (1\text{-}8)$$

On the other hand, PME is inhibited by PMEI, which is tip localized:

$$C_{PMEI} = k_{pmei}E \quad (1\text{-}9)$$

Since PME is inhibited by PMEI, the distribution of functional PME, or effective PME, is

$$C_{EP} = C_{PME} - C_{PMEI} \quad (1\text{-}10)$$

For simplification, the extensibility of hard pectin is considered to be 0 so that the extensibility of the cell wall (Φ) is determined by the percentage of soft pectin in the cell wall:

$$\Phi = \Phi_s P_s/(P_s + P_h) \quad (1\text{-}11)$$

Initial conditions:

$$P_s = 0, P_h = 0, s \in [-30, 30]$$

(ii) Cell Surface Extension

Assuming axisymmetric geometry of the cell, as the extensibility of the cell wall is known, the stresses and the strain rates of the cell wall in the meridional (subscript s) and the circumferential (subscript θ) directions (Supplementary Fig. 1d) are calculated using equations adopted from Dumais et al., 2006[1]. Here we made several assumptions to simplify the problem: (1) The yield stress of the cell wall is 0; (2) The cell wall is

isotropic; and (3) The normal stress and strain rate of the cell wall are negligible. Under these assumptions, we have:

Equilibrium equations

$$\sigma_s = \frac{p}{2\delta\kappa_\theta}, \tag{1-12}$$

$$\sigma_\theta = \frac{p}{2\delta\kappa_\theta}\left(2 - \frac{\kappa_s}{\kappa_\theta}\right), \tag{1-13}$$

Constitutive equations

$$\dot{\varepsilon}_s = \Phi(\sigma_s - \nu\sigma_\theta), \tag{1-14}$$

$$\dot{\varepsilon}_\theta = \Phi(\sigma_\theta - \nu\sigma_s), \tag{1-15}$$

Kinetic equations

$$\dot{\varepsilon}_s = v_n\kappa_s + \frac{\partial v_t}{\partial s}, \tag{1-16}$$

$$\dot{\varepsilon}_\theta = v_n\kappa_\theta + \frac{v_t\cos\varphi}{r}, \tag{1-17}$$

Computational Implementation

The algorithm for simulating pollen tube tip growth was implemented with MATLAB (The MathWorks, R2015b). See the last section for pseudocodes of the algorithm. Firstly, the meridian of the cell surface is discretized with n points. Given an initial stimulation of active ROP1 at the center of the cell meridian (the cell tip), the steady-state distribution of active ROP1 and exocytosis rate are obtained by solving Equation 1-1 to 1-5 using the finite difference method. With the exocytosis rate at each points, the distribution of pectins is computed using Equation 1-6 to 1-10 and the cell wall extensibility is computed using Equation 1-11. The stresses are calculated from the geometry, the cell wall thickness and the turgor pressure using the equilibrium equations (Equation 1-12, 1-13). With the extensibility and the stresses of the cell wall, the strain rates are obtained by the constitutive equations (Equation 1-14, 1-15). To determine the velocities of displacements from the strain rates, the kinetic equations (Equation 1-16, 1-17) are rearranged to give

$$v_\mathrm{n} = \frac{\dot{\varepsilon}_\theta}{\kappa_\theta} - Q\cos\varphi, \tag{1-18}$$

$$v_\mathrm{t} = Q\sin\varphi, \tag{1-19}$$

$$Q = \int \frac{\dot{\varepsilon}_s - \dot{\varepsilon}_\theta\kappa_s/\kappa_\theta}{\sin\varphi}\,\mathrm{d}s, \tag{1-20}$$

When the displacement velocities of each point on the cell surface are known, the position of each point after a small time interval, Δt, can be calculated. The new geometry of the cell surface is remeshed. To simulate the growth of the pollen tube during a period of time, T, the above procedure is repeated T/Δt times.

The Model of Pollen Tube Growth Guidance

The model of pollen tube guidance consists of three interconnecting parts, representing the major steps in pollen tube guidance: the perception of the diffusive guidance signal through the GEF-mediated positive feedback loop, the redistribution of the active ROP1 on the PM in response to the signal, and the changes in cell wall mechanics and cell morphology. The position and the shape of the cell, in turn, determine the local

concentration of the guidance signal along the cell periphery. The definitions and values of the parameters in this model are listed in Supplementary Table 4.

Guidance Signal Distribution and ROP1 Polarization

Firstly, we established the distribution of the guidance signal. Guidance signal molecules are constantly released from the ovules and diffuse over the surface of the medium with diffusion rate D_S and degradation rate k_S. At the stable stage, the distribution of the signal, S, is obtained by

$$D_{\mathrm{S}}\left(\frac{\partial^2 S}{\partial x^2}+\frac{\partial^2 S}{\partial y^2}\right)-k_{\mathrm{S}}S=0 \tag{2-1}$$

At the source of the guidance signal, the signal concentration is a constant, S_0.

The spatiotemporal distribution of exocytosis, E, and the active ROP1, R, in the guidance model are described by similar equations as in the tip growth model (see Equation 1-1 to 1-5), but on the three-dimensional surface of the PM:

$$E=k_{\mathrm{E}}R^{\alpha}, \tag{2-2}$$

$$\frac{\partial R}{\partial t}=k_{\mathrm{a}}R_{\mathrm{in}}\left(1-\frac{R}{R_{\mathrm{max}}}\right)-k_{\mathrm{d}}R+D\nabla_A^2 R \tag{2-3}$$

$$R_{\mathrm{in}}=R_{\mathrm{total}}-\int R\,\mathrm{d}A, \tag{2-4}$$

$$k_{\mathrm{a}}=k_{\mathrm{pf}}E, \tag{2-5}$$

$$k_{\mathrm{d}}=k_{\mathrm{nf}}k_{\mathrm{E}}, \tag{2-6}$$

∇_A^2 is the Laplace–Beltrami operator for the surface of the PM.

Guidance signal locally activates ROP1 through its positive regulator, GEF. Therefore, in the presence of the guidance signal, the local strength of the positive feedback of ROP1 is positively correlated with the signal concentration:

$$k_{\mathrm{pf}}=k_{\mathrm{pf0}}(1+S) \tag{2-7}$$

where k_{pf0} is the basal strength of the positive feedback when the guidance signal is absent.

Cell Wall Deformation

The cell wall of pollen tube is approximated as a thin layer of viscoplastic material[1]. For simplicity, we omitted the equations linking exocytosis to the distribution of pectins, and the extensibility of the cell wall is determined by the local exocytosis rate.

$$\Phi=\Phi_0 E \tag{2-8}$$

Using the thin shell theory, assuming the plane stress condition ($\sigma_z=\tau_{yz}=\tau_{zx}=0$), the strain rate field is

$$\dot{\boldsymbol{\varepsilon}}=\begin{Bmatrix}\dot{\varepsilon}_x\\ \dot{\varepsilon}_y\\ \dot{\gamma}_{xy}\end{Bmatrix}=\begin{Bmatrix}\dfrac{\partial\dot{u}}{\partial x}\\ \dfrac{\partial\dot{v}}{\partial x}\\ \dfrac{\partial u}{\partial y}+\dfrac{\partial v}{\partial x}\end{Bmatrix}+\begin{Bmatrix}-z\dfrac{\partial^2\dot{w}}{\partial x^2}\\ -z\dfrac{\partial^2\dot{w}}{\partial y^2}\\ -2z\dfrac{\partial^2\dot{w}}{\partial x\,\partial y}\end{Bmatrix} \tag{2-9}$$

where $\dot{\varepsilon}_x$ and $\dot{\varepsilon}_y$ are the axial strain rates, $\dot{\gamma}_{xy}$ is the shear strain rate, and $\dot{u}$, $\dot{v}$ and $\dot{w}$ are the displacement velocities.

The stress field is

$$\boldsymbol{\sigma} = \begin{Bmatrix} \sigma_x \\ \sigma_y \\ \tau_{xy} \end{Bmatrix} = \frac{1}{\Phi(1-\nu^2)} \begin{bmatrix} 1 & \nu & 0 \\ \nu & 1 & 0 \\ 0 & 0 & \frac{1-\nu}{2} \end{bmatrix} \begin{Bmatrix} \dot{\varepsilon}_x \\ \dot{\varepsilon}_y \\ \dot{\gamma}_{xy} \end{Bmatrix} \tag{2-10}$$

Where σ_x and σ_y are the axial stresses, τ_{xy} is the shear stress, and ν is the Poisson's ratio. The equilibrium equation is derived based on the Principle of Virtual Work (see Computational Implementation section below).

We have tested an alternative model in which the guidance signal bypasses the ROP1-exocytosis signaling and regulates the cell wall extensibility directly (Supplementary Fig. 5a). In this model instead of Equation (2-7) and (2-8), we have

$$\Phi = \Phi_0(E + \rho S)$$

where ρ is a coefficient indicating the significance of guidance signals comparing to the ROP-exocytosis pathway in affecting the cell wall extensibility. k_{pf} is a constant independent of the guidance signal concentration.

Computational Implementation

We used the Finite Element Method (FEM) to solve the equations for the cell wall mechanics numerically[2,3], and the model was computationally implemented in MATLAB. See the last section for pseudocodes of the algorithms.

First of all, the cell wall is discretized into triangular elements, interconnecting at vertices, the nodes. Each element is approximated as a thin plate and has its own local axes (x', y', z'), where z' is the normal to the plane of the element. The three nodes of an element are i, j and k. The local coordinates of node i is $[x_i, y_i, z_i]^{\mathrm{T}}$(note that $z_i = 0$). The local displacement vector of a point in the element during a small time interval, Δt, is

$$\boldsymbol{\varphi}' = \begin{Bmatrix} u' \\ v' \\ w' \end{Bmatrix} \tag{2-11}$$

where u', v' and w' are the displacements along the x', y' and z' axes, respectively. Define the local nodal displacement vector as

$$\mathbf{d'}_{\boldsymbol{i}} = \begin{Bmatrix} u'_i \\ v'_i \\ w'_i \\ \theta_{x'_i} \\ \theta_{y'_i} \end{Bmatrix}, \quad \mathbf{d'} = \begin{Bmatrix} \mathbf{d'}_{\boldsymbol{i}} \\ \mathbf{d'}_{\boldsymbol{j}} \\ \mathbf{d'}_{\boldsymbol{k}} \end{Bmatrix} \tag{2-12}$$

where

$$\theta_{x'} = \frac{\partial w'}{\partial x'}, \quad \theta_{y'} = \frac{\partial w'}{\partial y'}$$

(1) Shape functions

Each element has 15 degrees of freedom. We use linear expansions for the u' and v' displacements and a cubic expansion for the w' displacement:

$$\begin{Bmatrix} u' \\ v' \\ w' \end{Bmatrix} = \begin{bmatrix} 1 & x' & y' & 0 & 0 & 0 & 0 & 0 & 0 & 0 & 0 & 0 & 0 & 0 & 0 \\ 0 & 0 & 0 & 1 & x' & y' & 0 & 0 & 0 & 0 & 0 & 0 & 0 & 0 & 0 \\ 0 & 0 & 0 & 0 & 0 & 0 & 1 & x' & y' & x'^2 & y'^2 & x'^3 & x'^2y' & x'y'^2 & y'^3 \end{bmatrix} \begin{Bmatrix} a_1 \\ a_2 \\ a_3 \\ b_1 \\ b_2 \\ b_3 \\ c_1 \\ \vdots \\ c_9 \end{Bmatrix}$$

or

$$\boldsymbol{\varphi}' = \mathbf{Pa} \tag{2-13}$$

On the other hand,

$$\mathbf{d}'_i = \begin{bmatrix} 1 & x_i & y_i & 0 & 0 & 0 & 0 & 0 & 0 & 0 & 0 & 0 & 0 & 0 & 0 \\ 0 & 0 & 0 & 1 & x_i & y_i & 0 & 0 & 0 & 0 & 0 & 0 & 0 & 0 & 0 \\ 0 & 0 & 0 & 0 & 0 & 0 & 1 & x_i & y_i & {x_i}^2 & {y_i}^2 & {x_i}^3 & {x_i}^2 y_i & x_i {y_i}^2 & {y_i}^3 \\ 0 & 0 & 0 & 0 & 0 & 0 & 0 & 1 & 0 & 2x_i & 0 & 3{x_i}^2 & 2x_i y_i & {y_i}^2 & 0 \\ 0 & 0 & 0 & 0 & 0 & 0 & 0 & 0 & 1 & 0 & 2y_i & 0 & {x_i}^2 & 2x_i y_i & 3{y_i}^2 \end{bmatrix} \begin{Bmatrix} a_1 \\ a_2 \\ a_3 \\ b_1 \\ b_2 \\ b_3 \\ c_1 \\ \vdots \\ c_9 \end{Bmatrix}$$

$$\mathbf{d}' = \mathbf{Ca} \tag{2-14}$$

Therefore,

$$\boldsymbol{\varphi}' = \mathbf{PC}^{-1}\mathbf{d}' = \mathbf{Nd}', \;\; \mathbf{N} = \mathbf{PC}^{-1} \tag{\textbf{\textit{2-15}}}$$

where $\boldsymbol{N}$ is the shape function matrix.

(2) Kinematic equations

Assuming the plane stress condition ($\sigma_{z'} = \tau_{y'z'} = \tau_{z'x'} = 0$), the local strain rate field is

$$\dot{\boldsymbol{\varepsilon}}' = \begin{Bmatrix} \dot{\varepsilon}_{x'} \\ \dot{\varepsilon}_{y'} \\ \dot{\gamma}_{x'y'} \end{Bmatrix} = \frac{\partial}{\partial t} \begin{Bmatrix} \dfrac{\partial u'}{\partial x'} - z' \dfrac{\partial^2 w'}{\partial x'^2} \\ \dfrac{\partial v'}{\partial x'} - z' \dfrac{\partial^2 w'}{\partial y'^2} \\ \dfrac{\partial u'}{\partial y'} + \dfrac{\partial v'}{\partial x'} - 2z' \dfrac{\partial^2 w'}{\partial x' \partial y'} \end{Bmatrix} = \mathbf{S}\frac{\partial \boldsymbol{\varphi}'}{\partial t}, \mathbf{S} = \frac{\partial}{\partial t} \begin{bmatrix} \dfrac{\partial}{\partial x'} & 0 & -z' \dfrac{\partial^2}{\partial x'^2} \\ 0 & \dfrac{\partial}{\partial y'} & -z' \dfrac{\partial^2}{\partial y'^2} \\ \dfrac{\partial}{\partial y'} & \dfrac{\partial}{\partial x'} & -2z' \dfrac{\partial^2}{\partial x' \partial y'} \end{bmatrix}$$

Therefore,

$$\dot{\boldsymbol{\varepsilon}}' = \mathbf{B}\frac{\partial \mathbf{d}'}{\partial t}, \;\; \mathbf{B} = \mathbf{SN} \tag{2-16}$$

(3) Constitutive equations

The local stress field is

$$\boldsymbol{\sigma}' = \begin{Bmatrix} \sigma_{x'} \\ \sigma_{y'} \\ \tau_{x'y'} \end{Bmatrix} = \frac{1}{\Phi(1-\nu^2)} \begin{bmatrix} 1 & \nu & 0 \\ \nu & 1 & 0 \\ 0 & 0 & \frac{1-\nu}{2} \end{bmatrix} \begin{Bmatrix} \dot{\varepsilon}_{x'} \\ \dot{\varepsilon}_{y'} \\ \dot{\gamma}_{x'y'} \end{Bmatrix} = \mathbf{D}\dot{\boldsymbol{\varepsilon}}' \tag{2-17}$$

$$\mathbf{D} = \frac{1}{\Phi(1-\nu^2)} \begin{bmatrix} 1 & \nu & 0 \\ \nu & 1 & 0 \\ 0 & 0 & \frac{1-\nu}{2} \end{bmatrix} \tag{2-18}$$

(4) Equilibrium equations and the element stiffness equation

Based on the Principle of Virtual Work, we have

$$\frac{\partial U'}{\partial \mathbf{d}'} = \frac{\partial \Omega'}{\partial \mathbf{d}'} \tag{2-19}$$

Here U' is the virtual strain energy:

$$U' = \frac{1}{2}\iiint_V \boldsymbol{\sigma}'^{\mathrm{T}}\boldsymbol{\varepsilon}'\mathrm{d}V = \frac{1}{2}\iiint \dot{\boldsymbol{\varepsilon}}'^{\mathrm{T}}\mathbf{D}\boldsymbol{\varepsilon}'\mathrm{d}V = \frac{1}{2}\iiint \frac{\partial \mathbf{d}'^{\mathrm{T}}}{\partial t}\mathbf{B}^{\mathrm{T}}\mathbf{D}\mathbf{B}\mathbf{d}'\mathrm{d}V$$

and Ω' is the virtual work of external forces:

$$\Omega' = \iint_A \boldsymbol{\varphi}'^{\mathrm{T}}\begin{Bmatrix}0\\0\\p\end{Bmatrix}\mathrm{d}A$$

where p is the turgor pressure, V and A are the volume and the surface area of the cell wall, respectively. Therefore,

$$\iiint_V \frac{\partial}{\partial t}\mathbf{B}^{\mathrm{T}}\mathbf{D}\mathbf{B}\mathbf{d}'\mathrm{d}V = \iint_A \mathbf{N}^{\mathrm{T}}\begin{Bmatrix}0\\0\\p\end{Bmatrix}\mathrm{d}A \tag{2-20}$$

We define the local stiffness matrix **K**' and the local force vector **f**' as

$$\mathbf{K}' = \iiint_V \mathbf{B}^{\mathrm{T}}\mathbf{D}\mathbf{B}\mathrm{d}V,\ \ \mathbf{f}' = \iint_A \mathbf{N}^{\mathrm{T}}\begin{Bmatrix}0\\0\\p\end{Bmatrix}\mathrm{d}A \tag{2-21}$$

$$\mathbf{K}' = \begin{bmatrix}\mathbf{K}'_{ii} & \mathbf{K}'_{ij} & \mathbf{K}'_{ik}\\ \mathbf{K}'_{ji} & \mathbf{K}'_{jj} & \mathbf{K}'_{jk}\\ \mathbf{K}'_{ki} & \mathbf{K}'_{kj} & \mathbf{K}'_{kk}\end{bmatrix}, \mathbf{f}' = \begin{Bmatrix}\mathbf{f}'_i\\ \mathbf{f}'_j\\ \mathbf{f}'_k\end{Bmatrix}, \mathbf{f}'_i = \begin{Bmatrix}f_{x'_i}\\ f_{y'_i}\\ f_{z'_i}\\ m_{x'_i}\\ m_{y'_i}\\ m_{z'_i}\end{Bmatrix}$$

Then we have the element stiffness equation

$$\mathbf{K}'\frac{\partial \mathbf{d}'}{\partial t} = \mathbf{f}' \tag{2-22}$$

As the mesh is refined in this system, singularity of the stiffness matrix may arise due to the quasi-coplanar situation of adjacent elements[3]. To avoid the singularity, an arbitrary rotational stiffness $k_{\theta z}$ is inserted into the diagonal of the local stiffness matrix:

$$\bar{\mathbf{K}}'_{ii} = \begin{bmatrix}\mathbf{K}'_{ii} & \mathbf{0}\\ \mathbf{0} & k_{\theta_z}\end{bmatrix} \tag{2-23}$$

The degrees of freedom of each node becomes six instead of five:

$$\bar{\mathbf{d}}'_i = \begin{Bmatrix}\mathbf{d}'_i\\ \theta_{z'_i}\end{Bmatrix} = \begin{Bmatrix}u'_i\\ v'_i\\ w'_i\\ \theta_{x'_i}\\ \theta_{y'_i}\\ \theta_{z'_i}\end{Bmatrix}, \qquad \bar{\mathbf{f}}'_i = \begin{Bmatrix}\mathbf{f}'_i\\ 0\end{Bmatrix} = \begin{Bmatrix}f_{x'_i}\\ f_{y'_i}\\ f_{z'_i}\\ m_{x'_i}\\ m_{y'_i}\\ 0\end{Bmatrix}$$

Then we have $\theta_{z'i} = 0$. The new element stiffness equation becomes

$$\bar{\mathbf{K}}'\frac{\partial \bar{\mathbf{d}}'}{\partial t} = \bar{\mathbf{f}}' \tag{2-24}$$

(5) Coordinate transformation

The assembly of the global stiffness equation requires that the stiffness matrix, nodal displacement vector and force vector are defined in the same global coordinate system. Denote the unit vectors of the local coordinate system by *i*, *j* and *k*, then we have the transformation matrices

$$\mathbf{R} = [i, j, k], \qquad \mathbf{T}_i = \begin{bmatrix} \mathbf{R} & \mathbf{0} \\ \mathbf{0} & \mathbf{R} \end{bmatrix}, \qquad \mathbf{T} = \begin{bmatrix} \mathbf{T}_i & \mathbf{0} & \mathbf{0} \\ \mathbf{0} & \mathbf{T}_i & \mathbf{0} \\ \mathbf{0} & \mathbf{0} & \mathbf{T}_i \end{bmatrix}$$

So we have

$$\mathbf{d} = \mathbf{T}\bar{\mathbf{d}}' \tag{2-25}$$

$$\mathbf{f} = \mathbf{T}\bar{\mathbf{f}}' \tag{2-26}$$

where **f** is the element force vector in the global axes. Noting that **T** is orthogonal, we have

$$\mathbf{T}\bar{\mathbf{K}}'\mathbf{T}^{\mathrm{T}}\frac{\partial \mathbf{d}}{\partial t} = \mathbf{f} \tag{2-27}$$

Therefore, the element stiffness matrix in the global axes is

$$\mathbf{K} = \mathbf{T}\bar{\mathbf{K}}'\mathbf{T}^{\mathrm{T}} \tag{2-28}$$

(6) The global stiffness equation and boundary conditions

The global stiffness matrix and the global force vector are assembled in the standard manner in the FEM. The boundary of the cell wall is fixed:

$$u_i = v_i = w_i = \theta_{x_i} = \theta_{y_i} = 0$$

The displacement of each nodes on the cell surface determines the deformed geometry of the cell, and remeshing is performed by inserting new nodes to element edges that exceed a certain threshold. The above procedure is repeated to simulate continuous cell growth.

Pseudocodes for Computational Implementation

The Model of Pollen Tube Tip Growth

```
SET parameters
INITIALIZE cell wall coordinates by discretizing a circle with n nodes
INITIALIZE ROP1 at each node
INITIALIZE pectin at each node

COMUTE steady state ROP1 distribution (Equation 1-2)
      FOR each time step
            COMPUTE new ROP1 distribution by adding the amount of
            activation and deactivation
            COMPUTE new ROP1 distribution after diffusion by implicit
            finite difference
      END FOR
COMUTE exocytosis at each node (Equation 1-1)

FOR each time step

      COMPUTE effective PME at each node (Equations 1-8, 1-9, 1-10)
      COMPUTE secreted soft pectin and converted hard pectin
      UPDATE pectin at each node (Equations 1-6, 1-7)
```

```
      COMPUTE cell wall extensibility at each node (Equation 1-11)
      COMPUTE cell wall curvatures at each node from node coordinates
      COMPUTE cell wall stresses at each node (Equations 1-12, 1-13)
      COMPUTE cell wall strain rates at each node (Equations 1-14, 1-
      15)
      COMPUTE cell wall velocities at each node in normal and tangent
      axes (Equations 1-18, 1-19, 1-20)
      CONVERT cell wall velocities to the global coordinate system
      UPDATE cell wall node coordinates
      COMPUTE the advection of pectins from the tangential growth
      velocity
      UPDATE pectin at each node (Equations 1-6, 1-7)

      REDISCRETIZE the cell wall to ensure sufficient number of nodes
      at the growth region

END FOR
```

The Model of Pollen Tube Growth Guidance

```
SET parameters
COMPUTE the steady state of guidance signal distribution (Equation 2-1)
INITIALIZE ROP1 distribution at each node
COMPUTE exocytosis at each node (Equation 2-2)

DISCRETIZE a spherical initial cell surface by Delaunay triangulation
      GET coordinates of nodes and mapping between nodes and elements
PREALLOCATE vectors for storing coordinates of nodes in non-growing
region

FOR each time step dT

      FIND boundary nodes
      COMPUTE cell wall extensibility at each node from exocytosis

      PREALLOCATE system matrix and vector
      FOR each element
            GET global coordinates of the three nodes
            CONVERT global coordinates to local coordinates
            COMPUTE the coordinate transformation matrices
            COMPUTE the mean cell wall extensibility of the element
            COMPUTE element stiffness matrix and convert to global
            coordinates
            COMPUTE element force vector and convert to global
            coordinates
            UPDATE system matrix and vector
      END FOR
      COMPUTE displacement of each node by solving the global stiffness
      equation
      UPDATE node coordinates

      COMPUTE ROP1 distribution at each node by Finite Element Method
            ASSEMBLE system matrix and vector
            COMPUTE guidance signal concentration at each node
```

```
        COMPUTE ROP1 positive feedback rate at each node (Equation
        2-7)
        DIVIDE dT into smaller time steps dt
        FOR each time step dt
              COMPUTE new ROP1 distribution by adding the amount of
              activation and deactivation
              COMPUTE new ROP1 distribution after diffusion by
              solving the global stiffness equation
        END FOR
  COMUTE exocytosis at each node

  REFINE elements by adding new nodes to element edges with length
  exceeding a threshold
  REMOVE nodes in non-growing region

END FOR
```

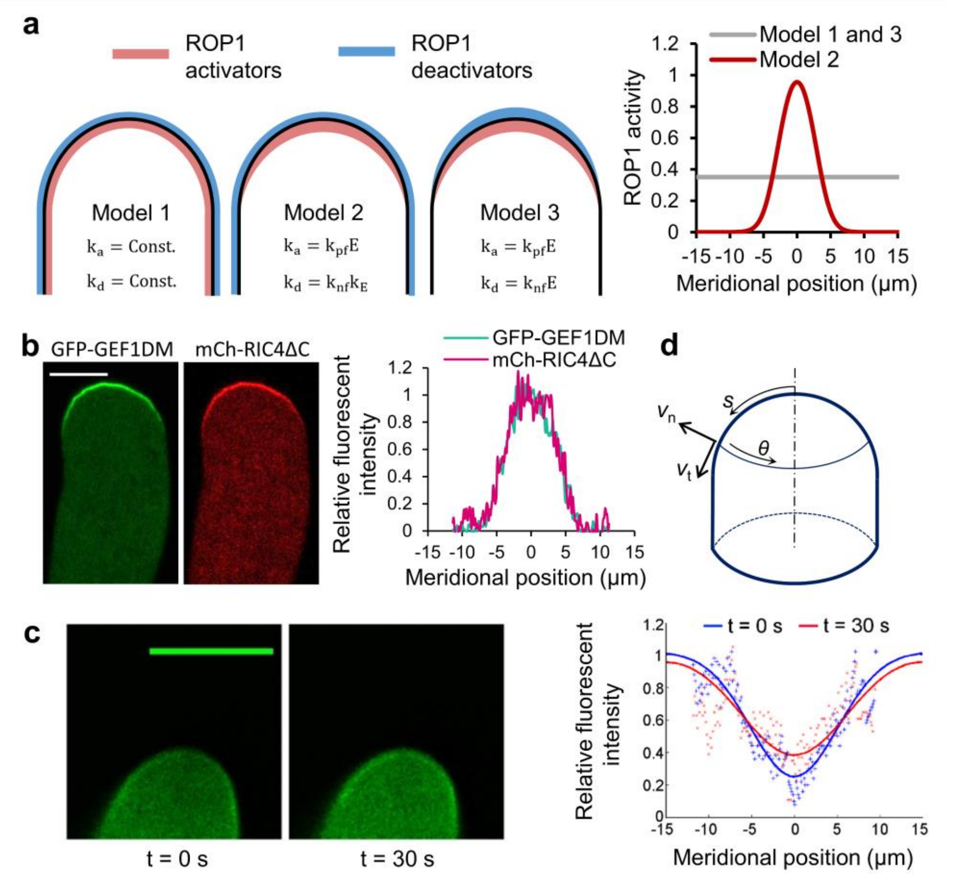

Supplementary Figure 1. Simulations and measurements to determine the structure and parameters of the model

(a) Testing different modes of feedbacks for ROP1 polarization. When the distribution of the activators and the deactivators of ROP1 are independent of exocytosis (model 1), the model is not able to generate polarized active ROP1. Active ROP1 polarizes when the activators are concentrated on the tip by local positive feedback through exocytosis and the deactivators are evenly distributed by global inhibition (model 2). With both local inhibition and local positive feedback (model 3), active ROP1 is not able to polarize either. **(b)** *Arabidopsis* RopGEF1 co-localized with RIC4ΔC in tobacco pollen tubes. Representative confocal images (left) and distribution curves (right) of GFP-GEF1DM (containing L103D and W246S mutations to disrupt GEF activity) and mCherry-RIC4ΔC transiently co-expressed in tobacco pollen tubes. Scale bar: 5 μm. **(c)** Measuring the diffusion constant of membrane ROP1 in pollen tubes by FRAP. *Arabidopsis* pollen tubes expressing GFP-ROP1 were treated by liquid pollen tube growth medium containing 20 nM Latrunculin B (LatB) for 10-15 min to completely disrupt exocytosis in order to measure the diffusion of ROP1 accurately. Control pollen tubes treated by 0.2% DMSO displayed no growth defects and were not shown. The cell membrane at the tip of pollen tubes is photobleached by high intensity 488 nm laser light, and time-lapse confocal images were taken for 30 s. Scale bar: 5 μm. The right panel shows the distribution of GFP-ROP1 along the cell periphery immediately after photobleaching (t = 0 s) and 30 s after photobleaching (t = 30 s). **(d)** Schematic showing the principal directions of an axis-symmetric pollen tube. *s*: the meridional direction; *θ*: the circumferential direction. The surface velocity is decomposed into components normal (v_n) and tangential (v_t) to the

surface. Since the tangential velocity on the circumferential direction is 0, it is equivalent to velocity in the meridional direction.

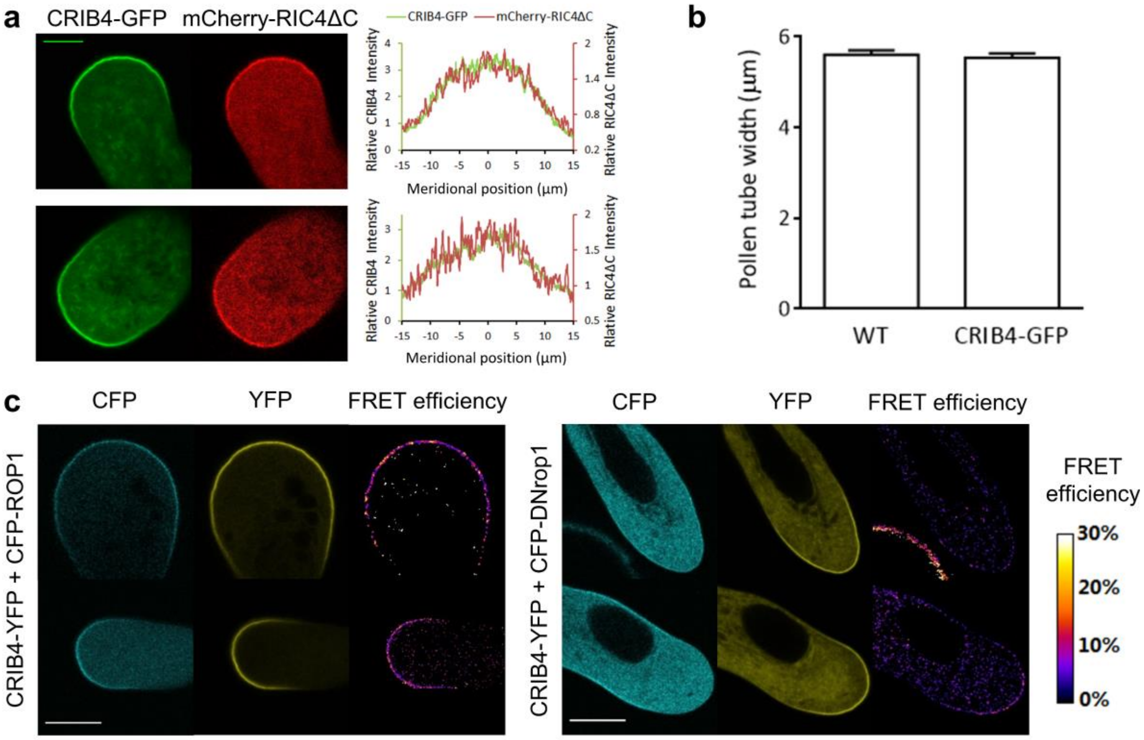

Supplementary Figure 2. CRIB4-GFP is a specific marker for active ROP1
Previously we used GFP-RIC4ΔC as an active ROP1 marker in tobacco pollen tubes[2], but expression of this marker in *Arabidopsis* caused growth depolarization. We found that further deletion outside of the CRIB motif[1] (termed CRIB4, residues 64-130) from RIC4 eliminated the depolarization effect, but retains the ability of CRIB4 to interact with active ROP1. **(a)** CRIB4-GFP co-localizes with mCherry-RIC4ΔC in tobacco pollen tubes. Scale bar: 5 μm. **(b)** Expression of CRIB4-GFP in *Arabidopsis* pollen tubes does not affect the shape of pollen tubes. Wild-type: n = 23, CRIB4-GFP: n = 21. Error bars show s.e.m.. P = 0.58 (Student's t-test). **(c)** FRET between CRIB4-YFP and CFP-ROP1/DNrop1 shows that CRIB4 preferentially binds with active ROP1. Scale bar: 5 μm. Data were representative of 4 samples (left panel) or 6 samples (right panel). Thus we conclude that CRIB4-GFP is an accurate reporter for the ROP1 activity distributed to the apical PM of pollen tubes.

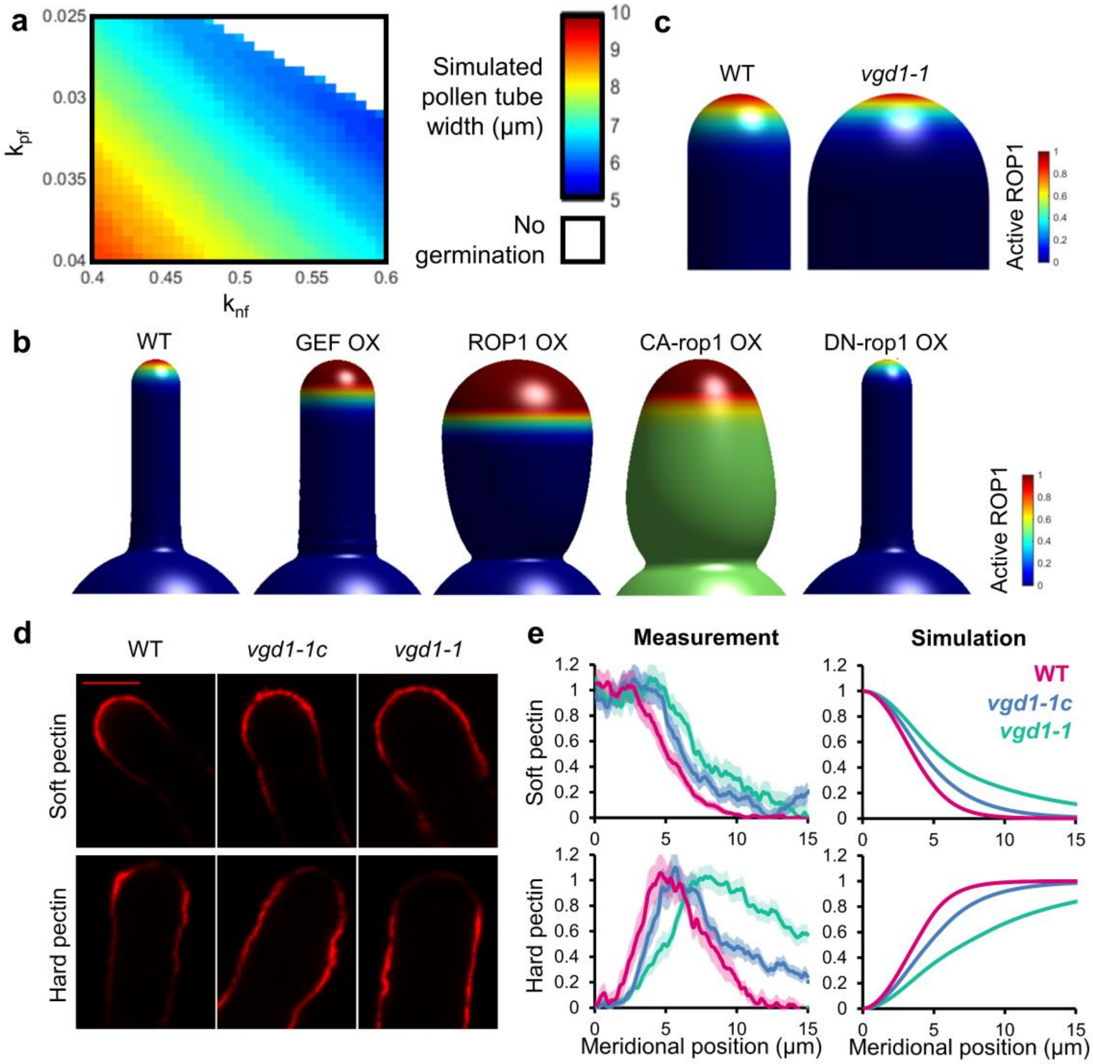

Supplementary Figure 3. The shape of pollen tubes depends on active ROP1 polarity and pectin distribution.

(a) Altering k_{pf} (active ROP1 positive feedback rate) and k_{nf} (active ROP1 negative feedback rate) results in pollen tubes with different width. **(b)** Simulations of other reported ROP1 signaling mutants. Overexpressing of RopGEF is simulated by increasing the positive feedback strength, which increased the width of pollen tubes. Overexpressing ROP1 or CA-rop1 causes depolarization of pollen tubes, which is simulated by increasing the total amount of ROP1 (ROP1 OX) or increasing the background concentration of active ROP1 and decreasing the negative feedback rate (CA-rop1 OX), since CA-rop1 competes with endogenous ROP1 for the GAPs. Overexpressing of DN-rop1 is simulated by decreasing the positive feedback strength, since DN-rop1 competes with endogenous ROP1 for the GEFs, and the simulated pollen tube shows increased cell width and lower growth rate. Color indicates the level of active ROP1 on the PM. **(c-e)** Simulation and measurements of pollen tubes that are deficient in pectin de-esterification. **(c)** Simulation of wild-type and *vgd1-1* pollen tubes. Only the tip region of pollen tubes was shown. Color indicates the level of active ROP1 on the PM. **(d)** Confocal microscopy images of pectin immunostaining of wild-type, *vgd1-1* and *vgd1-1c* (*vgd1-1* partially complemented

with LAT52::VGD1-GFP) pollen tubes with JIM7 and JIM5. Scale bar: 5 µm. **(e)** Observed and simulated distribution of pectins in wild-type, *vgd1-1* and *vgd1-1c* pollen tubes. Error bars show s.e.m..

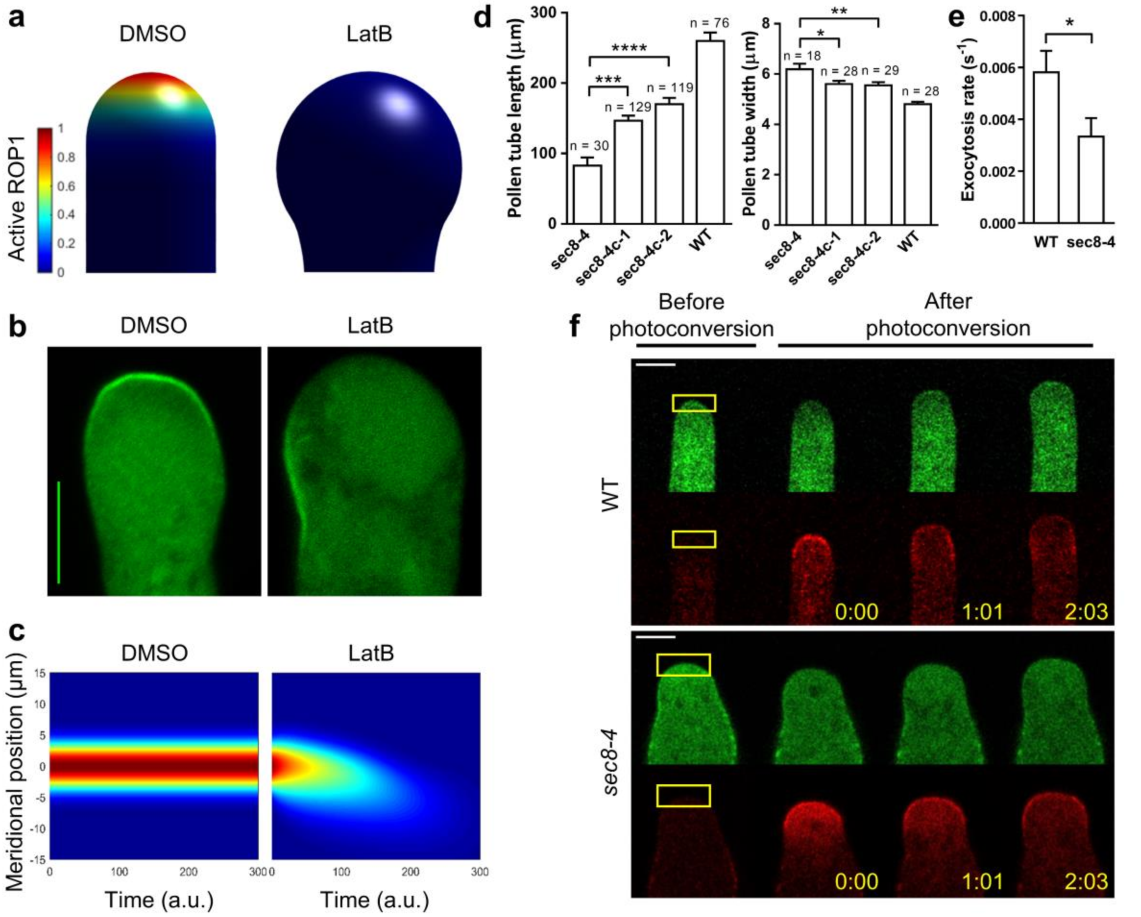

Supplementary Figure 4. Simulation of pollen tubes defective in exocytosis.
(a) Simulation of DMSO- or LatB-treated pollen tubes. Only the tip region of pollen tubes was shown. Color indicates the level of active ROP1 on the PM. **(b)** Confocal microscopy images of DMSO- or LatB-treated pollen tubes expressing CRIB4-GFP. Scale bar: 5 µm. Images are representatives of 5 DMSO- and 5 LatB-treated pollen tubes. **(c)** Simulated shifting of active ROP1 to one side of the cell assuming that exocytosis rate is asymmetrically reduced if the impact of LatB on exocytosis is not uniform. Color indicates the level of active ROP1 on the PM (see **(a)** for color bar). **(d)** The defects in pollen tube morphology of *sec8-4* were partially complemented by the expression of LAT52::GFP-SEC8. *sec8-4c*-1 and -2 are two independent complementation lines of *sec8-4*. Error bars show s.e.m.. * $P \leq 0.05$, *** $P \leq 0.001$, **** $P \leq 0.0001$ (Student's t-test). **(e-f)** Measuring the exocytosis rate of *sec8-4* pollen tubes by Corrected Fluorescence Recovery after Photoconversion (cFRAPc). **(e)** Average exocytosis rate of wild-type (n=15) and *sec8-4* (n=15) pollen tubes. Error bars show s.e.m.. * $P \leq 0.05$ (Student's t-test). **(f)** Representative cFRAPc images of wild-type and *sec8-4* pollen tubes expressing PRK1-Dendra2. In each panel both green and red channels were shown. Yellow boxes indicate the photoconverted regions. Numbers show time after photoconversion (min:sec). Scale bar: 5 µm.

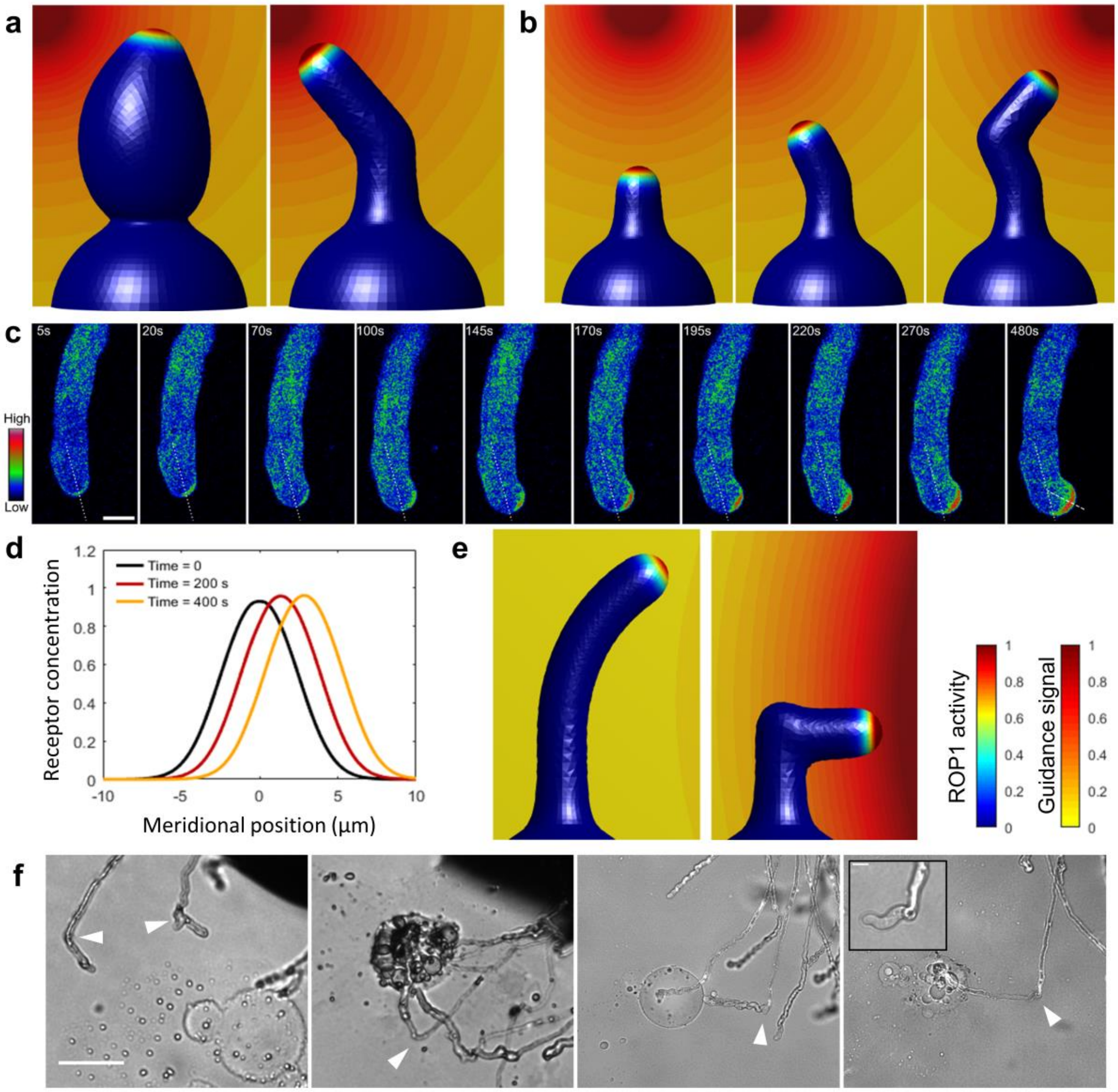

Supplementary Figure 5. Pollen tube guidance is mechanistically linked with tip growth.

(a) Assuming the guidance signal affects cell wall extensibility through a pathway independent of the ROP-exocytosis signaling network, the model is not able to simulate normal pollen tube morphology during guidance. Though the growth is asymmetric, the cell is depolarized. **(b)** The integration of guidance signal into the ROP1-exocytosis network reproduces normal pollen tube guidance. The pollen tube turns to follow the direction of the guidance signal gradient. Color on the surface of the pollen tube shows the concentration of active ROP1; Color in the background shows the concentration of the guidance signal (see color bar in e). **(c)** Confocal image sequence showing local wall thickening preceding the reorientation of the pollen tube attracted by an AtLURE1-containing gelatin bead. Color bar indicates the intensity of fluorescence. Numbers show time after the beginning of imaging. Dashed lines show the direction of growth. Scale bar: 5 μm. **(d)** Assuming that the receptor of the guidance signal is targeted to the PM by exocytosis, the model reproduces the redistribution of the receptor on the PM towards the

guidance signal source. **(e)** Simulated pollen tube maintains normal cell shape and constant cell width in either shallow (left) or steep (right) gradients of guidance signal. **(f)** Dramatic sharp turnings of pollen tubes observed in the semi-*in-vitro* assay with AtLURE1-containing gelatin beads. Arrowheads indicate the turnings. Scale bar: 100 μm (inset scale bar: 10 μm).

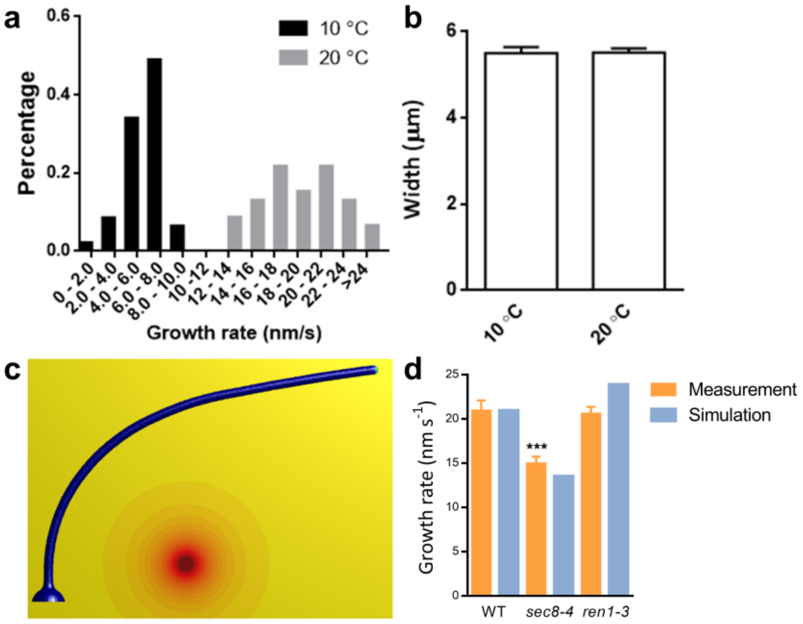

Supplementary Figure 6. The model predicts the optimal conditions for efficient guidance.
(a-b) Testing the guidance efficiency of pollen tubes with different growth rates by culturing pollen tubes at different temperatures. **(a)** Histogram showing the distributions of pollen tube growth rates at 10°C and at 20°C do not overlap. **(b)** The average width of pollen tubes at 10°C (n = 24) or 20°C (n = 36) has no significant difference. Error bars show s.e.m.. P = 0.94 (Student's t-test). **(c)** Simulation shows that when the pollen tube growth rates are beyond a certain threshold, the pollen tube will miss the target completely. **(d)** Predicted growth rate of wild-type and mutant pollen tubes by the model comparing to measurement. Growth rates predicted by the model are standardized by the wild-type value. Average growth rates were measure from 10 wild-type, 23 *sec8-4* and 31 *ren1-3* pollen tubes. Error bars show s.e.m.. *** P ≤ 0.001 (Student's t-test).

Supplementary Table 1. Sensitivity analysis of simulated pollen tube width to model parameters.

Parameter	Sensitivity
α	-2.60
k_{E}	-0.55
R_{max}	-0.32
k_{pme}	-0.32
R_{total}	-0.32
D	0.28
k_{pf}	-0.26
k_{pmei}	-0.20
δ	0.07
p	-0.07
Φ_{s}	-0.07
k_{sec}	0.06
k_{nf}	-0.03

Supplementary Table 2. Parameter setting for the Exocytosis-ROP1 Polarization module

Variables	Definition	Unit
s	Meridional position on the PM	μm
t	Time	s
R	The concentration of active ROP1 in the PM	1
R_{in}	The concentration of inactive ROP1 in the cytoplasm	1
k_{a}	ROP1 activation coefficient	1 s^{-1}
k_{d}	ROP1 deactivation coefficient	1 s^{-1}
E	Exocytosis rate	1
**Constants**	**Definition**	**Value**
$R_{\max}$	Maximum active ROP1 concentration on the PM	5
R_{total}	Total ROP1 (including active and inactive ROP1)	30
D	Diffusion coefficient of ROP1 on the PM	0.2 $\mu m^2\ s^{-1}$
k_{E}	Coefficient linking ROP1 activity with exocytosis rate	1
α	Coefficient representing the degree of nonlinearity in the positive feedback	1.25
k_{pf}	Strength of exocytosis-mediated positive feedback	0.0280 s^{-1}
k_{nf}	Strength of exocytosis-mediated negative feedback	0.5081 s^{-1}

Supplementary Table 3. Parameter setting for the Exocytosis-Wall Extension module

Variables	Definition	Unit
s	Meridional position on the PM	μm
t	Time	min
C_{PME}	The concentration of PME on the PM	1
C_{PMEI}	The concentration of PMEI on the PM	1
P_{s}	The concentration of soft (methyl esterified) pectin	1
P_{h}	The concentration of hard (de-esterified) pectin	1
E	Exocytosis rate	1
Φ	Relative cell wall extensibility	1 MPa^{-1} min^{-1}
E_{t}	Exocytosis rate at the tip, $\mathrm{E}(\mathrm{s}=0)$	1
σ_s	Normal stress on the meridional direction	MPa
σ_θ	Normal stress on the circumferential direction	MPa
κ_s	Curvature of the cell wall on the meridional direction	1 $\mu\mathrm{m}^{-1}$
κ_θ	Curvature of the cell wall on the circumferential direction	1 $\mu\mathrm{m}^{-1}$
$\dot{\varepsilon}_s$	Strain rate on the meridional direction	1 min^{-1}
$\dot{\varepsilon}_\theta$	Strain rate on the circumferential direction	1 min^{-1}
v_n	Local velocity normal to the cell surface	μm min^{-1}
v_t	Local velocity tangential to the cell surface	μm min^{-1}
r	Cross-sectional radius of the cell	μm
φ	The angle between the normal to the surface and the cell axis	rad
**Constants**	**Definition**	**Value**
k_{pmei}	Coefficient linking exocytosis rate with the concentration of PMEI	0.9
k_{sec}	Coefficient of soft pectin secretion by exocytosis	0.1 min^{-1}
k_{pme}	Pectin de-esterification rate	0.8 min^{-1}
Φ_{s}	Extensibility of soft pectin	20 MPa^{-1} min^{-1}
p	Turgor pressure	0.2 MPa [4]
δ	Thickness of the cell wall	0.2 μm [5]
ν	The Poisson's ratio	0.5

Supplementary Table 4. Parameter setting for the model of pollen tube guidance

Variables	Definition	Unit
t	Time	min
x, y	Horizontal and vertical position parallel to the medium surface	μm
z	Position on the direction perpendicular to the medium surface	μm
S	Concentration of the guidance signal	1
R	The concentration of active ROP1 in the PM	1
R_{in}	The concentration of inactive ROP1 in the cytoplasm	1
k_{a}	ROP1 activation coefficient	$1\ s^{-1}$
k_{d}	ROP1 deactivation coefficient	$1\ s^{-1}$
k_{pf}	The local rate of exocytosis-mediated positive feedback	1
Φ	Relative cell wall extensibility	$1\ MPa^{-1}\ min^{-1}$
u, v, w	Displacements along the x, y and z axes	μm
$\sigma_x, \sigma_y, \sigma_z$	Stresses along the x, y and z axes	MPa
$\dot{\varepsilon}_x, \dot{\varepsilon}_y, \dot{\varepsilon}_z$	Strain rates along the x, y and z axes	$1\ min^{-1}$
**Constants**	**Definition**	**Value**
S_0	Concentration of the guidance signal at the signal source	1
D_{S}	Diffusion coefficient of the guidance signal on the medium	$10\ \mu m^2\ s^{-1}$
k_{S}	Degradation rate of the guidance signal	$0.1\ s^{-1}$
k_{pf0}	Coefficient linking positive feedback rate to the concentration of guidance signal	$0.03\ s^{-1}$
k_{nf}	Rate of exocytosis-mediated negative feedback	$0.5\ s^{-1}$
R_{total}	Total ROP1 (including active and inactive ROP1)	100
α	Coefficient representing the degree of nonlinearity in the positive feedback	1.25
R_{max}	Maximum active ROP1 concentration in the PM	5
D	Diffusion coefficient of ROP1 in the PM	$0.5\ \mu m^2\ s^{-1}$
Φ_0	Coefficient linking active ROP1 to cell wall extensibility	$10^{-3}\ MPa^{-1}\ s^{-1}$
ν	The Poisson's ratio	0.5
p	Turgor pressure	0.2 MPa
δ	Thickness of the cell wall	0.2 μm [5]

Supplementary References

1. Dumais, J., Shaw, S. L., Steele, C. R., Long, S. R. & Ray, P. M., An anisotropic-viscoplastic model of plant cell morphogenesis by tip growth. *Int J. Dev Biol* **50**, 209-222 (2006).
2. Logan, D., *A First Course in the Finite Element Method*, 5th ed. (Stamford, CT : Cencage Learning, 2012).
3. Onate, E., *Structural Analysis with the Finite Element Method. Volume 2, Beams, Plates and Shells*. (Dordrecht ; London : Springer, 2013).
4. Benkert, R., Obermeyer, G. & Bentrup, F. W., The turgor pressure of growing lily pollen tubes. *Protoplasma* **198**, 1-8 (1997).
5. Chebli, Y., Kaneda, M., Zerzour, R. & Geitmann, A., The cell wall of the Arabidopsis pollen tube--spatial distribution, recycling, and network formation of polysaccharides. *Plant Physiol* **160**, 1940-1955 (2012).
