# Supplementary material for: Exocytosis-coordinated mechanisms for tip growth underlie pollen tube growth guidance
Source: Nat Commun. 2017 Nov 22;8:1687. doi: 10.1038/s41467-017-01452-0 (PMC5698331; doi:10.1038/s41467-017-01452-0)
Supplement: Supplementary file 2 — Description of Additional Supplementary Files [file 41467_2017_1452_MOESM2_ESM.pdf]

Description of Supplementary Files

File Name: Supplementary Movie 1
Description: **Simulation of pollen tube tip growth** Showing the distribution of active ROP1, cell wall extensibility, effective stress and areal strain rates during tip growth of a pollen tube simulated by the model. The pollen tube is germinated from a sphere representing the pollen grain.

File Name: Supplementary Movie 2
Description: **Simulation of pollen tube growth following a guidance signal gradient** Showing the distribution of active ROP1 and the cell morphology during pollen tube guidance simulated by the model. The active ROP1 cap and the growth direction of the pollen tube follows the changing direction of the guidance signal gradient. Scale bar: 5 µm.

File Name: Supplementary Movie 3
Description: **Confocal time series of a pollen tube making turnings in semi-*in-vitro* assay** *Arabidopsis* pollen tubes expressing CRIB4-GFP were observed to make multiple turnings towards AtLURE1-containing gelatin beads in the semi-*in-vitro* assay. Arrowheads indicate the peak of the CRIB4-GFP on the PM, which always predicts the future growing direction. Numbers show time after the beads were placed (min:sec). Scale bar: 5 µm.
